# Global and regional burden of chronic respiratory disease in 2016 arising from non-infectious airborne occupational exposures: a systematic analysis for the Global Burden of Disease Study 2016

**DOI:** 10.1136/oemed-2019-106013

**Published:** 2020-02-13

**Authors:** GBD 2016 Occupational Chronic Respiratory Risk Factors Collaborators, Tim Driscoll

**Keywords:** occupational exposure, COPD, occupational asthma, pneumoconiosis, work

## Abstract

**Objectives:**

This paper presents detailed analysis of the global and regional burden of chronic respiratory disease arising from occupational airborne exposures, as estimated in the Global Burden of Disease 2016 study.

**Methods:**

The burden of chronic obstructive pulmonary disease (COPD) due to occupational exposure to particulate matter, gases and fumes, and secondhand smoke, and the burden of asthma resulting from occupational exposure to asthmagens, was estimated using the population attributable fraction (PAF), calculated using exposure prevalence and relative risks from the literature. PAFs were applied to the number of deaths and disability-adjusted life years (DALYs) for COPD and asthma. Pneumoconioses were estimated directly from cause of death data. Age-standardised rates were based only on persons aged 15 years and above.

**Results:**

The estimated PAFs (based on DALYs) were 17% (95% uncertainty interval (UI) 14%–20%) for COPD and 10% (95% UI 9%–11%) for asthma. There were estimated to be 519 000 (95% UI 441,000–609,000) deaths from chronic respiratory disease in 2016 due to occupational airborne risk factors (COPD: 460,100 [95% UI 382,000–551,000]; asthma: 37,600 [95% UI 28,400–47,900]; pneumoconioses: 21,500 [95% UI 17,900–25,400]. The equivalent overall burden estimate was 13.6 million (95% UI 11.9–15.5 million); DALYs (COPD: 10.7 [95% UI 9.0–12.5] million; asthma: 2.3 [95% UI 1.9–2.9] million; pneumoconioses: 0.58 [95% UI 0.46–0.67] million). Rates were highest in males; older persons and mainly in Oceania, Asia and sub-Saharan Africa; and decreased from 1990 to 2016.

**Conclusions:**

Workplace exposures resulting in COPD, asthma and pneumoconiosis continue to be important contributors to the burden of disease in all regions of the world. This should be reducible through improved prevention and control of relevant exposures.

**Key messages**

**What is already known about this subject?**

- Occupational respiratory exposures have been shown to be an important cause of chronic work-related respiratory disease at national and global level.
- The last analysis of this issue at the global level was for the year 2000—this paper provides a new analysis for 2016.

**What are the new findings?**

- Analysis of the Global Burden of Disease data set suggests that globally there were about 519,100 deaths and 13.6 million disability-adjusted life years in 2016 from chronic respiratory disease due to occupational airborne exposures.
- The population attributable fraction for chronic obstructive pulmonary disease (COPD) was 17% and for asthma was 10%.
- Workplace exposures resulting in COPD, asthma and pneumoconioses remain important contributors to the burden of disease in all regions of the world.

**How might this impact on policy or clinical practice in the foreseeable future?**

- These findings highlight opportunities to continue to reduce chronic respiratory disease burden worldwide by improving prevention and control of workplace airborne exposures.

## Introduction

Airborne respiratory hazards (inorganic and organic particulate matter, vapours, gases and fumes) are a common exposure in occupational settings and many studies have identified resulting malignant and chronic respiratory disease as an important component of the occupational injury and disease burden at both country and global levels.^1–10^ Work-related respiratory diseases remain a problem even in high-income countries, as shown by incident cases of pneumoconioses that are still occurring.^11 12^

The Global Burden of Disease (GBD) Comparative Risk Assessment (CRA) project was the first to consider the burden of occupational chronic respiratory disease comprehensively at a regional and global level, estimating the burden for the year 2000.^13^ That study included airborne exposures leading to asthma, chronic obstructive pulmonary disease (COPD), asbestosis, coal workers’ pneumoconiosis (CWP) and silicosis.^14^

The new GBD initiative, conducted by the Institute of Health Metrics and Evaluation, first focused on 2010^15^ and has been updated several times since.^16–19^ It provides a detailed analysis of the burden of disease and injury overall and arising from specific risk factors. One set of those risk factors comprises occupational exposures,^18^but no detailed analysis of the occupational risk factor results has been undertaken.

This paper presents a detailed analysis of the global and regional burden of chronic respiratory disease arising from non-infectious occupational airborne exposures, as estimated in the GBD 2016 study. Malignant occupational respiratory disease^20^ and an overview of all occupational risk factors^21^ are considered in companion papers.^20^

## Methods

### General approach

The general methodology used in GBD 2016 is described elsewhere,^18^ as is the overall approach to occupational risk factors.^21^ These methods are briefly summarised here. A more detailed description is provided here of the analyses of occupational exposures to particulate matter, gases and fumes (PMGF), secondhand smoke (SHS), asthmagens and pneumoconiotic dusts and their associated outcomes.

The burden of occupational respiratory disease for PMGF and SHS (causing COPD) and for asthmagens was estimated using the population attributable fraction (PAF), that is, the proportion of deaths or disability-adjusted life years (DALYs) that would not have occurred if exposure was at the theoretical minimum risk exposure level; this was then used to estimate attributable numbers of deaths or DALYs. The PAF requires information on the relative risk of the disease due to the exposure of interest and the proportion of the target population exposed. Pneumoconioses were estimated directly as part of the overall GBD estimates of prevalence and deaths for each included cause. Age-standardised rates (per 100 000 people) were based only on persons aged 15 and above. Results were calculated for all years from 1990 to 2016, inclusive; the 2016 findings are the focus of this paper. The socio-demographic index (SDI) is a composite indicator of development status based on total fertility rate, mean education for those aged 15 and older and lag distributed income per capita.^18^Region-specific, SDI-specific and global results are reported here. Country-specific information is available through the GBD Compare data visualisation.^22^ High-income countries were defined as countries in the Australasia, high-income North America, Western Europe and Asia Pacific regions, and low/middle-income (LMI) countries as all other countries. Employment data came from the International Labour Organization Labour Force,^23^ supplemented where necessary by sub-national data sources and modelling. PAFs for all carcinogens except asbestos were estimated for each age-sex-country group using the equation based on Levin^24^:

$$PAF=\frac{\sum_{x=1}^{n}RR\left(x\right)P\left(x\right)-1}{\sum_{x=1}^{n}RR\left(x\right)P\left(x\right)}$$

where P*(*x) is the proportion of persons exposed at level x in the relevant population and RR(x) is the relative risk corresponding to exposure level x.

### PMGF and SHS

Industry was used as a proxy for exposure to PMGF because we identified no suitable and valid data sources at a country or global level of exposure to PMGF, either singly or to PMGF as a group. Current industry was used as the basis of exposure estimates, but the estimates of proportions exposed (ie, workers who experienced more than trivial exposure) within each industry (nine categories—see online supplementary table S1) were designed to take into account past exposure (to estimate ever exposed), given that both past and current exposure appear to increase the risk of COPD. Estimates of proportion exposed at lower and higher levels in high income and LMI countries were based on sparse published data (see online supplementary material) and expert opinion by GBD collaborators (online supplementary table S1). Information on risk was obtained by conducting a systematic review of international literature and meta-analysis (unpublished) of relevant results.^5 25^ Relative risks in these studies were for COPD greater than or equal toGlobal Initiative for Chronic Obstructive Lung Disease(GOLD) stage II: defined as requiring non-reversibility after using bronchodilators for provocation, a forced expiratory volume in one second/forced vital capacity (FEV_1_/FVC) ratio of less than 0.70 and an FEV_1_ of less than 80% predicted.^26^ Relative risk estimates were used for an overall ‘lower’ level (RR=1.44; 95% CI 1.07–1.95) and an overall ‘higher’ level (RR=2.31; 95% CI 1.45 to 3.73) of exposure to the agents of concern (‘higher’ and ‘lower’ were based on the exposure descriptions in the papers).^5 25^ The reference group was persons not working and persons working in trade, finance or service industries. The prevalence of exposure to PMGF was determined using the following equation:

10.1136/oemed-2019-106013.supp1 Supplementary data

$$\begin{array}{ll} 1)PrevalenceofExposure_{c,y,s,a,l}= & \sum_{EA}{Proportion}_{EAC,y}*EAP_{c,y,s,}\\ & *Exposurelevelproportion_{EAl} \end{array}$$

where EAP = economically active population, c = country, s = sex, EA = economic activity, l = level of exposure, y =year and a = age.

Exposure information on SHS was based on the CAREX (Carcinogen Exposure) database, which provides industry-specific information from 1990 to 1993 on the prevalence of exposure to various carcinogens in countries of Western Europe,^27^ as described elsewhere.^20^ The relevant relative risks were those used for SHS in the general GBD 2016 analysis.^18^

### Asthmagens

Exposure and relative risks for asthmagens were based on the current occupation distribution (eight categories—see online supplementary table S2) because there were no suitable and valid data sources at a country or global level describing exposure to the wide range of occupational asthmagens. All relative risk information, except that for agricultural occupations, came from a study by Karjalainen and coworkers, a comprehensive national population study of incident asthma.^7 8^ Relative risks for agricultural occupations were based on a study by Kogevinas and coworkers,^28^ using a weighted average of the separate estimates for ‘farmers’ and ‘agricultural’ workers provided in the paper. This information was used because the results were thought to be more generalisable to agriculture in the rest of the world, especially for LMI regions. Separate risks were available and used for males and females (except for agricultural operations), although the sex-specific risks were similar and within the limits of random variation. The same relative risks were used for all age groups. The counterfactual was persons not working and administrative workers. Byssinosis was included as asthma for the purposes of the analysis. The prevalence of exposure to asthmagens was determined using the following equation:

$$\begin{array}{l} 2)PrevalenceofExposure_{c,y,s,a}=\sum_{EA}{Proportion}_{occc,y}*EAP_{c,y,s,} \end{array}$$

where EAP = economically active population, c = country, s = sex, OCC = occupation, y = year and a = age.

### Pneumoconiotic dusts

As mentioned, pneumoconioses were estimated directly as part of the overall GBD estimates of prevalence and deaths for each included cause, rather than using the attributable fraction approach. The methods used are described elsewhere.^29^ The attributable fraction is essentially 100% because virtually all pneumoconioses arise as a result of occupational exposure. Separate estimates were available for silicosis, asbestosis and CWP, with the remaining cases grouped under an ‘other pneumoconiosis’ category.^23^

### Statistical approach

The main modelling and analyses employed to produce the GBD 2016 data, and the calculation and use of 95% uncertainty intervals (95% UI), were as described elsewhere.^18 21^ Uncertainty intervals are primarily presented in detail in the tables to assist with the flow of the text.

## Results

There were estimated to be about 519 000 (95% UI 441 000–609 000) deaths from chronic respiratory disease in 2016 due to occupational airborne risk factors. The vast majority (460 000 [95% UI 382 000–551 000]; 89%) of these were due to COPD arising from PMGF and SHS. The remaining deaths were from asthma (37 600 [95% UI 28 400–47 900]; 7%), due to exposure to a range of asthmagens, and from pneumoconiosis (21 500 [95% UI 17 900–25 400]; 4%), arising from exposure to pneumoconiotic dusts. Males accounted for 75% (390 000) of the deaths overall and between 69% (asthmagens) and 88% (pneumoconiotic dusts) for individual risk factors. The relative contribution of the different risk factors was similar when the burden was measured in terms of DALYs (13.6 [95% UI 11.9–15.5] million DALYs overall; COPD: 10.7 [95% UI 9.0–12.5] million; asthma: 2.3 [95% UI 1.9–2.9] million; pneumoconioses: 0.58 [95% UI 0.49–0.67] million), with 79% of the DALYs due to PMGF and SHS. Males accounted for 73% (9.9 million) of the DALYs (table 1).

**Table 1:** Global occupational-attributable deaths, DALYs and PAFs from chronic respiratory disease due to airborne exposures by risk factor and sex, 2016 (number, percent and proportion [95% uncertainty interval])*

Risk factor	Total	Deaths	Males‡	%§	Males	DALYs	Total	%¶	Males	PAFs†	Total
		Females				Females				Females	
Asthmagens	26 103	11 471	37 574	7.2	1 468 347	871 133	2 339 480	17.2	13.0	7.1	9.9
	(17 900–35 300)	(8700–15 200)	(28 400–47 900)		(1 141 200–1 874 300)	(666 100–1 109 600)	(1 860 900–2 923 300)		(12.0–14.0)	(6.3–8.0)	(9.0–11.0)
PMGF+SHS**	343 122	116 958	460 080	88.6	7 969 986	2 717 967	10 687 953	78.6	21.0	11.0	17.0
	(270 900–422 000)	(87 100–153 300)	(381 500–551 300)		(6 518 000–9 469 200)	(2 143 400–3 328 600)	(9 019 900–12 517 000)		(18.0–25.0)	(9.0–13.0)	(14.0–20.0)
Pneumoconiotic dusts	18 997	2491	21 488	4.1	518 917	58 060	576 977	4.2	100	100	100
	(15 500–22 700)	(2100–3200)	(17 900–25 400)		(439 900–611 300)	(49 400–70 700)	(493 600–673 500)				
Total	388 222	130 920	519 142	**100.0**	9 957 269	3 647 170	13 604 438	**100.0**	**19.0**	**9.0**	**15.0**
	(313 900–466 600)	(100 500–167 700)	(441 000–608 900)		(8 520 200–11 450 300)	(3 010 800–4 335 200)	(11 912 000–15 502 800)		(16.0–22.0)	(7.0–11.0)	(13.0–17.0)

*DALYs=disability-adjusted life years; PAF=population attributable fraction.

†PAFs (%) based on DALYs.

‡The numbers in brackets in the whole table are 95% uncertainty intervals.

§Percentage of chronic respiratory disease deaths due to occupational risk factors that were due to this risk factor.

¶Percentage of chronic respiratory disease DALYs due to occupational risk factors that were due to this risk factor.

**Particulate matter, gases and fumes (PMGF) and secondhand smoke (SHS) causing chronic obstructive pulmonary disease.

### PMGF and SHS

The PAF for COPD arising from occupational exposures was 17% (95% UI 14%–20%) for DALYs (16% for deaths), ranging from a low of 10% in Central sub-Saharan Africa to 21% in East Asia (table 2). The PAF was much higher in males (21%) than females (11%) and peaked at about 24% in 60–64-year-old males. The highest number of deaths and the highest rate of deaths from COPD due to occupational exposures occurred in the older age groups, often beyond usual retirement age. Males had three to four times the number and rate of deaths compared with females. The peak for number of DALYs occurred in the 65–74 year age group, but the rate of DALYs increased considerably with age and was highest in the 75–84 year group for both males and females (online supplementary figures S1-S4).

**Table 2:** Deaths, YLLs, YLDs, DALYs and PAFs from COPD due to occupational exposure to PMGF and SHS, by region, 2016 (number, percent, rate, proportion)*

Region	Deaths	Deaths	YLLs	YLDs	DALYs	DALYs	Deaths	YLLs	YLDs	DALYs	YLL	YLD	PAF	PAF
	number	%	number	number	number	%	rate†	rate†	rate†	rate†	%‡	%‡	(deaths)§	(DALYs)¶
High-income North America	16 669	3.6	287 084	154 716	441 800	4.1	9.8	173	95	267	65.0	35.0	9.4	11.9
Australasia	1047	0.2	15 975	4922	20 897	0.2	7.7	122	39	161	76.4	23.6	10.0	13.1
High-income Asia Pacific	5150	1.1	61 981	70 847	132 828	1.2	4.8	61	76	136	46.7	53.3	12.0	15.5
Western Europe	18 258	4.0	250 656	73 485	324 141	3.0	7.8	113	35	148	77.3	22.7	9.1	11.6
Southern Latin America	2844	0.6	44 526	4967	49 493	0.5	10.8	174	20	194	90.0	10.0	12.2	14.3
Eastern Europe	6391	1.4	119 566	21 018	140 584	1.3	6.3	119	21	140	85.0	15.0	12.7	13.6
Central Europe	4622	1.0	78 183	20 299	98 482	0.9	7.9	136	36	172	79.4	20.6	12.2	13.9
Central Asia	2584	0.6	51 612	8277	59 889	0.6	10.1	190	29	219	86.2	13.8	16.4	16.8
Central Latin America	7042	1.5	107 850	25 554	133 404	1.2	9.3	139	31	171	80.8	19.2	13.6	15.1
Andean Latin America	1241	0.3	17 903	4217	22 120	0.2	7.1	99	23	122	80.9	19.1	15.3	16.1
Caribbean	1191	0.3	20 636	3576	24 213	0.2	7.2	126	22	147	85.2	14.8	13.2	14.2
Tropical Latin America	9936	2.2	179 699	25 150	204 849	1.9	13.7	239	32	271	87.7	12.3	15.1	17.3
East Asia	171 300	37.2	2 634 163	592 605	3 226 768	30.2	31.5	461	99	560	81.6	18.4	18.8	20.5
Southeast Asia	28 029	6.1	508 878	350 306	859 183	8.0	15.0	252	164	415	59.2	40.8	18.3	18.7
Oceania	886	0.2	21 518	3182	24 700	0.2	35.9	767	107	874	87.1	12.9	13.9	13.1
North Africa and Middle East	9834	2.1	193 777	69 764	263 541	2.5	6.9	125	42	167	73.5	26.5	12.4	12.8
South Asia	157 117	34.1	3 049 410	1 176 092	4 225 502	39.5	34.8	626	227	853	72.2	27.8	15.9	16.5
Southern sub-Saharan Africa	2117	0.5	39 719	15 450	55 169	0.5	11.2	197	72	269	72.0	28.0	12.7	12.3
Western sub-Saharan Africa	4775	1.0	105 149	39 099	144 248	1.3	7.9	144	49	192	72.9	27.1	16.2	15.6
Eastern sub-Saharan Africa	7716	1.7	154 524	46 238	200 761	1.9	12.3	217	60	277	77.0	23.0	19.4	18.3
Central sub-Saharan Africa	1333	0.3	27 051	8328	35 379	0.3	7.3	130	36	166	76.5	23.5	9.8	9.5
*High SDI*	41 455	9.0	630 080	319 949	950 029	8.9	7.7	122	65	187	66.3	33.7	9.8	12.4
*High-middle SDI*	55 985	12.2	855 050	280 400	1 135 450	10.6	12.6	191	63	253	75.3	24.7	15.7	17.4
*Middle SDI*	185 785	40.4	3 067 814	1 016 253	4 084 066	38.2	24.0	377	110	486	75.1	24.9	17.9	19.3
*Low-middle SDI*	156 339	34.0	2 995 849	971 878	3 967 727	37.1	29.5	522	169	691	75.5	24.5	15.9	16.2
*Low SDI*	20 517	4.5	421 068	129 612	550 681	5.2	16.2	291	83	374	76.5	23.5	15.2	14.9
Global	460 080	100.0	7 969 861	2 718 092	10 687 953	100.0	18.1	309	103	412	74.6	25.4	15.7	16.8

*YLL=years of life lost; YLD=years of life lived with disability; DALYs=disability-adjusted life years; PAF=population attributable fraction; COPD=chronic obstructive pulmonary disease; PMGF=particulate matter, gases and fumes; SHS=secondhand smoke.

†Per 100 000 persons.

‡Percentage of DALYs.

§Percentage of all COPD deaths due to occupational exposures.

¶Percentage of all COPD DALYs due to occupational exposures.

SDI, socio-demographic index.

By far the highest number of deaths and DALYs from COPD occurred in East Asia and South Asia, the regions with the largest populations, which together accounted for about 71% of both measures. The highest rates of deaths were in Oceania, South Asia and East Asia, the rates in these three regions being considerably higher than elsewhere (the lowest rates were in high-income Asia Pacific and Eastern Europe).

The same regions had the highest DALY rates (the lowest DALY rates were in Andean Latin America, high-income Asia Pacific and the Caribbean). Rates tended to be higher in low-middle and middle SDI regions, and there was considerable variation between regions. Seventy-five percent of the DALYs were due to years of life lost (YLLs); this predominance of YLLs was seen in nearly all regions (table 2). Information on the separate contribution of SHS to COPD is presented in the online supplementary material.

### Asthmagens

The PAF for asthma from occupational exposures was estimated to be 9.9% (95% UI 9.0%–10.9%) based on DALYs (8.9% [95% UI 7.8–10.1%] based on deaths), ranging from 4.1% in Central sub-Saharan Africa to 12.0% in South Asia (table 3). The PAF was higher for males (13%) than females (7%) and peaked at around 18% between the ages of about 35 and 49 years.

**Table 3:** Deaths, YLLs, YLDs, DALYs and PAFs from asthma due to occupational exposure to asthmagens, by region, 2016 (number, percent, rate, proportion)*

Region	Deaths	Deaths	YLLs	YLDs	DALYs	DALYs	Deaths	YLLs	YLDs	DALYs	YLL	YLD	PAF	PAF
	number	%	number	number	number	%	rate†	rate†	rate†	rate†	%‡	%‡	(deaths)§	(DALYs)¶
High-income North America	388	1.0	15 269	71 605	86 873	3.7	0.3	10.6	50.2	60.7	17.6	82.4	8.8	10.7
Australasia	23	0.1	857	11 291	12 148	0.5	0.2	7.4	100.1	107.5	7.1	92.9	4.3	9.0
High-income Asia Pacific	135	0.4	3493	34 431	37 923	1.6	0.2	4.2	46.3	50.5	9.2	90.8	2.6	8.0
Western Europe	182	0.5	6525	94 309	100 834	4.3	0.1	3.6	55.8	59.4	6.5	93.5	2.8	8.4
Southern Latin America	46	0.1	1558	12 849	14 407	0.6	0.2	6.2	51.6	57.8	10.8	89.2	4.9	7.5
Eastern Europe	204	0.5	6750	34 060	40 810	1.7	0.2	7.2	38.1	45.2	16.5	83.5	5.2	8.2
Central Europe	59	0.2	1877	16 773	18 650	0.8	0.1	3.6	34.7	38.3	10.1	89.9	3.4	8.2
Central Asia	166	0.4	5423	13 953	19 376	0.8	0.6	17.5	42.9	60.4	28.0	72.0	8.0	9.4
Central Latin America	185	0.5	6315	32 529	38 844	1.7	0.2	7.0	34.3	41.4	16.3	83.7	5.7	6.8
Andean Latin America	31	0.1	952	8977	9929	0.4	0.2	4.8	42.0	46.7	9.6	90.4	7.4	6.8
Caribbean	140	0.4	5049	8442	13 491	0.6	0.8	29.6	49.3	78.9	37.4	62.6	7.5	6.5
Tropical Latin America	176	0.5	6291	22 647	28 937	1.2	0.2	7.5	26.7	34.2	21.7	78.3	6.3	7.2
East Asia	1164	3.1	35 597	162 558	198 156	8.5	0.2	5.7	26.6	32.3	18.0	82.0	5.1	9.3
Southeast Asia	7315	19.5	224 974	142 208	367 182	15.7	3.3	96.0	57.8	153.8	61.3	38.7	9.5	11.0
Oceania	292	0.8	9862	2619	12 481	0.5	9.3	297.6	71.8	369.5	79.0	21.0	7.6	8.5
North Africa and Middle East	1344	3.6	47 971	84 044	132 015	5.6	0.7	25.3	41.3	66.6	36.3	63.7	6.0	6.7
South Asia	21 085	56.1	669 102	223 370	892 472	38.1	3.9	117.5	37.9	155.4	75.0	25.0	9.7	12.0
Southern sub-Saharan Africa	474	1.3	17 146	13 274	30 420	1.3	2.0	69.4	49.5	118.9	56.4	43.6	7.7	8.0
Western sub-Saharan Africa	1395	3.7	50 249	51 856	102 105	4.4	1.5	50.7	45.3	96.1	49.2	50.8	10.7	8.5
Eastern sub-Saharan Africa	2452	6.5	85 585	75 354	160 939	6.9	2.9	91.8	68.2	160.0	53.2	46.8	13.2	10.7
Central sub-Saharan Africa	316	0.8	11 315	10 173	21 487	0.9	1.2	41.1	33.1	74.1	52.7	47.3	5.1	4.1
*High SDI*	*776*	*2.1*	27 685	213 996	241 681	*10.3*	*0.2*	*6.5*	*52.1*	*58.6*	*11.5*	*88.5*	*4.6*	*9.2*
*High-middle SDI*	1320	*3.5*	44 449	185 336	229 785	*9.8*	*0.3*	*9.1*	*38.1*	*47.2*	*19.3*	*80.7*	*6.2*	*9.1*
*Middle SDI*	8807	*23.4*	277 589	316 569	594 158	*25.4*	*1.0*	*30.5*	*34.5*	*65.0*	*46.7*	*53.3*	*8.1*	*9.3*
*Low-middle SDI*	21 524	*57.3*	683 953	297 559	981 512	*42.0*	*3.3*	*101.3*	*41.4*	*142.7*	*69.7*	*30.3*	*9.7*	*11.1*
*Low SDI*	5147	*13.7*	178 483	113 860	292 343	*12.5*	*2.9*	*94.3*	*52.3*	*146.5*	*61.1*	*38.9*	*9.9*	*8.9*
Global	37 574	100.0	1 212 160	1 127 320	2 339 480	100.0	1.4	44.0	40.5	84.5	51.8	48.2	8.9	9.9

*YLL=years of life lost; YLD=years of life lived with disability; DALYs=disability-adjusted life years; PAF=population attributable fraction.

†Per 100 000 persons.

‡Percentage of DALYs.

§Percentage of all asthma deaths due to occupational exposures.

¶Percentage of all asthma DALYs due to occupational exposures.

SDI, socio-demographic index.

Deaths arising from occupational exposure to asthmagens occurred at all ages from 15 to 79 years, but the highest numbers occurred in persons aged 55–64 and the highest rates in persons aged 65–79. The burden was spread more evenly across age groups in terms of DALYs, with the highest number of DALYs in the 45–54 year age group and the highest rates in the 55–64 year age group (online supplementary figures S5-S8).

The highest number of deaths occurred in South Asia and Southeast Asia, and rates were highest in the low and low-middle SDI regions, particularly Oceania, South Asia and Southeast Asia (the lowest rates were in Western Europe and Central Europe). A similar pattern was seen for DALYs (the lowest rates were in East Asia and Tropical Latin America). Overall, YLLs and years of life with disability each contributed about 50% to the DALYs. However, low and low-middle SDI regions had a much higher proportion of DALYs due to YLLs compared with high-income regions, reflecting that a higher proportion of deaths occurred at younger ages in these regions compared with the high and high-middle SDI regions (table 3).

## Pneumoconiotic dusts

The PAF for all pneumoconioses was assumed to be 100%. Silicosis (48%) was the largest specific cause of death from pneumoconiosis, ahead of asbestosis (16%) and CWP (12%), but about one-quarter of the deaths were classified in the ‘other pneumoconiosis’ category. There was a similar distribution between pneumoconiosis categories in terms of DALYs (table 4). The number of deaths increased with age until age 85 years and over, and the age-standardised death rates were highest in the older age groups. There was a broader distribution of DALYs across age groups, and although the rates still increased with increasing age, the rate was highest in the 75–84 year age group (online supplementary figures S9-S12).

**Table 4:** Global occupational-attributable deaths and DALYs from pneumoconioses due to exposure to pneumoconiotic dusts, by region, 2016 (number and rate)*

Region	Deaths Asb*	Deaths CWP*	Deaths Sil*	Deaths Oth*	Deaths total	%	Death rate†	YLLs %‡	YLDs‡	DALYs	DALYs %	DALY rate†
High-income North America	674	283	118	78	1153	5.4	0.7	55.6	44.4	27 309	4.7	16.5
Australasia	87	1	11	6	105	0.5	0.8	88.4	11.6	1444	0.3	10.8
High-income Asia Pacific	345	279	486	477	1587	7.4	1.5	95.9	4.1	21 657	3.8	21.4
Western Europe	948	330	1388	147	2814	13.1	1.2	92.2	7.8	35 385	6.1	15.5
Southern Latin America	30	17	132	18	197	0.9	0.7	87.9	12.1	3236	0.6	12.5
Eastern Europe	37	29	35	172	272	1.3	0.3	56.5	43.5	8501	1.5	8.6
Central Europe	38	75	83	64	260	1.2	0.4	48.3	51.7	9058	1.6	16.0
Central Asia	14	3	2	49	68	0.3	0.3	63.9	36.1	2460	0.4	8.7
Central Latin America	38	33	90	75	235	1.1	0.3	49.5	50.5	9294	1.6	11.4
Andean Latin America	9	7	20	82	117	0.5	0.7	91.3	8.7	2351	0.4	12.6
Caribbean	4	2	5	13	24	0.1	0.1	61.9	38.1	739	0.1	4.4
Tropical Latin America	65	50	235	117	468	2.2	0.6	85.0	15.0	12 780	2.2	16.1
East Asia	345	960	6443	1080	8828	41.1	1.5	66.5	33.5	303 318	52.6	49.8
Southeast Asia	37	13	39	119	208	1.0	0.1	24.8	75.2	16 480	2.9	7.7
Oceania	5	1	7	17	30	0.1	1.4	86.0	14.0	816	0.1	29.6
North Africa and Middle East	69	43	93	195	401	1.9	0.3	62.7	37.3	13 882	2.4	8.2
South Asia	547	519	1089	1953	4109	19.1	0.9	90.8	9.2	92 603	16.0	18.4
Southern sub-Saharan Africa	133	12	51	64	260	1.2	1.3	92.6	7.4	6142	1.1	28.9
Western sub-Saharan Africa	10	3	5	38	57	0.3	0.1	74.8	25.2	2099	0.4	2.4
Eastern sub-Saharan Africa	47	19	55	108	229	1.1	0.4	89.6	10.4	5745	1.0	7.5
Central sub-Saharan Africa	12	6	15	33	66	0.3	0.4	90.0	10.0	1680	0.3	7.5
*High SDI*	2015	*977*	1855	*770*	5617	*26.1*	*1.0*	*78.2*	*21.8*	89 690	*15.5*	*17.3*
*High-middle SDI*	*307*	*324*	1702	*569*	2902	*13.5*	*0.6*	*59.2*	*40.8*	90 287	*15.6*	*18.9*
*Middle SDI*	*598*	*778*	5277	1521	8173	*38.0*	*1.0*	*68.6*	*31.4*	273 759	*47.4*	*31.7*
*Low-middle SDI*	*485*	*548*	1440	1781	4253	*19.8*	*0.8*	*83.4*	*16.6*	109 444	*19.0*	*18.2*
*Low SDI*	*90*	*57*	*129*	*266*	*542*	*2.5*	*0.4*	*88.6*	*11.4*	13 797	*2.4*	*8.8*
**Global**	3495	2685	10 402	4906	21 488	100.0	0.8	71.9	28.1	576 977	100.0	21.9

*YLL=years of life lost; YLD=years of life lived with disability; DALYs=disability-adjusted life years; PAF=population attributable fraction; Asb’=asbestosis; CWP=coal workers’ pneumoconiosis; ‘Sil’=silicosis; ‘Oth’=other pneumoconiosis.

†Per 100 000 persons.

‡Percentage of DALYs.

SDI, socio-demographic index.

The highest number of deaths and DALYs overall and for silicosis and CWP occurred in East Asia, South Asia and Western Europe, with high-income North America replacing East Asia for asbestosis deaths. The age-standardised death rates were highest in high-income Asia Pacific, East Asia and Oceania (the lowest rates were in Southeast Asia and the Caribbean), and the DALY rates highest in East Asia, Oceania and Southern sub-Saharan Africa (the lowest rates were in the Caribbean and Southeast Asia). Sixty-two percent of the silicosis deaths and 36% of the CWP deaths occurred in East Asia, and 27% of the asbestosis deaths occurred in Western Europe, which also had the second-highest rate (behind East Asia) of silicosis deaths. Western Europe, South Asia and East Asia had the highest number of asbestosis deaths, and East Asia, Australasia and Western Europe had the highest rate of asbestosis deaths (table 4—the rate data for individual pneumoconioses are not shown here).

### Changes over time

For COPD, there was little change (4% rise) in the number of deaths due to occupational exposure to PMGF and SHS between 1990 and 2016, but the (standardised) rate of death from COPD declined by 41% over this time. For asthmagens, the number of deaths due to occupational exposure increased by 7% and the rate of death declined by 36%. The number of deaths from pneumoconioses changed minimally (1%) over this period, but the rate of death from pneumoconioses declined by 41%. Changes in the numbers and rates of DALYs were similar to those seen for deaths, except for asthma, which had a 27% increase in DALYs between 1990 and 2016. The PAFs for asthma rose considerably over this time (21% for deaths; 28% for DALYs), but there was little change in the PAFs for COPD (table 5).

**Table 5:** Change in global occupational-attributable deaths and DALYs from chronic respiratory disease due to occupational exposure to asthmagens, PMGF, SHS and pneumoconiotic dusts between 1990 and 2016, number and per capita (number and percent [95% uncertainty interval])*

Risk factor	Deaths			DALYs		
	1990†	2016	% change	1990	2016	% change
Asthmagens	35 228	37 574	6.7	1 845 494	2 339 480	26.8
	(*24 103–48 462*)	(*28 362–47 936*)	*(−19.5* to *36.1*)	(*1 406 629–2 327 424*)	(*1 860 896–2 923 319*)	(*0.8* to *58.4*)
PMGF+SHS	441 702	460 080	4.2	9 825 539	10 687 953	8.8
	(*367 000–521 000*)	(*381 500–551 300*)	*(−13.6* to *24.8*)	(*8 149 400–11 533 600*)	(*9 019 900–12 517 000*)	(*−8.2* to *27.4*)
Pneumoconiotic dusts	21 209	21 488	1.3	567 941	576 977	1.6
	(*16 000–31 400*)	(*17 900–25 400*)	*(−15.6* to *19.8*)	(*442 576–832 555*)	(*493 632–673 528*)	(*−13.1* to *18.6*)
Total	498 139	519 142	4.2	12 238 974	13 604 410	11.2
	(*407 000–600 900*)	(*427 800–624 600*)	*(−14.9* to *25.4*)	(*10 667 700–13 881 800*)	(*11 912 000–15 502,800*)	(*−2.7* to *26.7*)

Risk factor	Deaths per 100 000 persons			DALYs per 100 000 persons		
	1990†	2016	% change	1990	2016	% change
Asthmagens	2.2	1.4	−36.0	108	85	−21.4
	(*1.5–* *3.0*)	(*1.0–* *1.8*)	(*−51.7* to *−18.1*)	(*82*–*136*)	(*67*–*106*)	(*−37.5* to *−1.7*)
PMGF+SHS	31.0	18.2	−41.3	653	410	−37.1
	(*26.8–* *36.5*)	(*15.0–* *21.9*)	(*−51.7* to *−29.4*)	(*545*–*768*)	(*346*–*482*)	(*−47.1* to *−26.1*)
Pneumoconiotic dusts	1.43	0.84	−41.3	36	22	−39.7
	(*1.08* *–* *2.10*)	(*0.70* *–* *0.99*)	(*−51.0* to *−30.8*)	(*28*–*53*)	(*19*–*26*)	(*−48.5* to *−29.7*)
Total	34.6	20.4	−40.9	796	517	−35.1
	(*28.4–* *41.6*)	(*16.7* *–* *24.6*)	(*−* *51.6* to *−* *28.8*)	(*655*–*957*)	(*431*–*613*)	(*−45.8* to *−23.0*)

Risk factor	Population attributable fraction (deaths) (%)			Population attributable fraction (DALYs) (%)		
	1990*	2016	% change	1990	2016	% change
Asthmagens	7.4 (*6.1–* *8.8*)	8.9 *(7.8–* *10.1*)	20.6 (*5.4–* *36.0*)	7.7 (*6.8–* *8.8*)	9.9 (*9.0–* *10.9*)	27.6 (*16.0–* *40.3*)
PMGF+SHS	16.3	15.7	−3.6	16.4	16.9	2.5
	(*13.7–* *19.1*)	(*13.8–* *18.6*)	(*−20.0* to *14.0*)	(*14.0–* *19.0*)	(*14.3–* *19.7*)	(*−13.0* to *19.8*)

*DALYs=disability-adjusted Life Years; PMGF=particulate matter, gases and fumes; SHS=secondhand smoke.

†The numbers in brackets in the whole table are 95% uncertainty intervals.

## Discussion

This analysis of the GBD 2016 study has shown there is a considerable burden of chronic respiratory disease worldwide and in all regions arising from exposure to occupational risk factors. Chronic obstructive pulmonary disease is the primary resulting disease, in terms of both deaths and DALYs, but asthma and pneumoconioses are also important. Rates were much higher in males than females for all these disorders, but important in both. The lower female rates reflect the fact that women are less likely to be employed in tasks that involve the relevant exposures.^23^ The results are consistent with those from the overall GBD respiratory analysis.^30^ The decreases in per capita burden for most measures, and the increase for asthma DALYs, result primarily from changes in the relevant PAFs that, in turn, reflect changes in the occupation and industry distribution, which are the basis of the exposure assessments.

### PMGF, SHS and COPD

The global estimate of the PAF for COPD arising from occupational exposure to PMGF and SHS (17% for DALYs; 16% for deaths) is consistent with most published findings for individual countries and overall. These have typically reported PAFs of the order of 10%–15%, although much higher values have been estimated, particularly for non-smokers, typically due to differences in the level or type of exposures of the included subjects or the use of different assumptions.^1 10 31–35^ In addition, as smoking rates diminish, the PAF for occupational risk factors will increase. In comparison, the GBD 2016 study estimated PAFs for COPD in regard to smoking and SHS of 43% and ambient particulate matter pollution of 27%.^22^ The CRA study (covering the year 2000) estimated 318 000 deaths and PAFs from occupational exposure of 13% based on DALYs and 12% based on deaths.^14^ The Burden of Obstructive Lung Disease (BOLD) study documented a direct relationship between COPD prevalence and number of years worked in dusty jobs.^36^

### Asthmagens and asthma

As with COPD related to occupational exposures, the occupational asthma PAF estimates of 10% for DALYs and 9% for deaths from this study are consistent with most published findings for individual countries, which are of the order of 10%–15%^3 7 8 10 28^ and comparable to the PAF due to smoking (10% for DALYs; 14% for deaths).^22^ The CRA study, which was based on the year 2000, estimated a PAF of 11% based on DALYs and 17% based on deaths (and estimated 38 000 deaths),^14^ the differences primarily arising from changes in the employment distribution and slight differences in the general methodology.^22^

### Pneumoconiotic dusts and pneumoconioses

Obtaining reliable global information on pneumoconiosis cases is challenging. This analysis identified silicosis as the predominant pneumoconiosis, with much lower numbers of cases of asbestosis and CWP. The increase in rates with age is consistent with the published literature,^37^ and the number of deaths is consistent with the publicly available data for many countries, but also differs considerably for some others for which the estimates here are notably different from the numbers reported in the WHO Mortality Database.^38^ The reason for this is not clear, but presumably is because of the use of different primary data sources and assumptions in the GBD modelling process. It is likely that most of the moderate proportion of pneumoconiosis deaths and DALYs (both 23%) coded in GBD 2016 as due to ‘Other pneumoconioses’ were actually due to silicosis, asbestosis or CWP, as these have always been identified as the three main pneumoconioses. The different coding is likely to have arisen due to incomplete coding in the source data and the way this was allocated to specific categories.

### Methodological considerations and limitations

Most of the methodological issues specific to the three main outcomes of interest have already been considered in the relevant sections of the Discussion. The main general uncertainties have been considered in detail in the companion overview paper.^21^ Issues of particular relevance to the presented analysis included basing exposure prevalence estimates on industry (for PMGF and SHS) and occupation (for asthmagens); uncertainty in the prevalence and level of exposure to PMGF overall and in different industries; the potential for mismatch between the relative risk estimates used and the exposure circumstances to which they have been applied; not explicitly taking into account the potential effect of differences in smoking habits and environmental exposures between regions and over time; probable heterogeneity in terms of how chronic respiratory conditions are identified, diagnosed and managed worldwide; and not including some potentially relevant risk factors and outcomes such as respiratory infections,^39^ other occupational causes of fibrosis apart from pneumoconioses and lung disease arising from nanoparticle exposure.^40^ For both COPD and asthma, the extent and effect of any mismatch between the exposure and the relative risk estimate applied in LMI countries are not clear. It would be helpful to have usable information on this from LMI countries, which might allow different risk estimates to be applied in these countries if appropriate. However, currently the necessary data are not available.

### Implications and uses of the data

The main finding of this study is that workplace exposures resulting in COPD, asthma and pneumoconioses remain important contributors to the burden of disease in all regions of the world. The relevant exposures are respiratory and it should be possible to minimise all (or most), and in some instances to essentially eliminate them, through appropriate commitment to, and implementation of, exposure control interventions to decrease the airborne exposure levels of the relevant hazards. However, it must be recognised that there are a range of PMGF implicated as increasing the risk of COPD and hundreds of known occupational asthmagens. Elimination or appropriate control of many of these exposures will take considerable resources and effort and requires continued vigilance. The study does not provide information on the cost or practicality of eliminating or better controlling the relevant exposures, and the results for COPD and pneumoconioses largely reflect past exposures. However, the high burden of COPD cases suggests the relevant exposures should be a priority in the area of occupational airborne exposures resulting in chronic respiratory disease. The findings also have implications for healthcare costs and social protection in older individuals. Finally, further investment in country-level data sources, especially in LMI countries, would help improve the accuracy and usefulness of the estimates generated by the GBD study.

### Conclusions

There are many respiratory conditions that can arise directly, or indirectly, from work. The results from this study indicate that non-malignant/non-infectious respiratory diseases arising from occupational exposures are an important cause of death and disability worldwide. Many of these cases should be preventable by adopting better health and safety approaches, particularly through improved engineering and working conditions.

10.1136/oemed-2019-106013.supp2 Supplementary data
