# Supplementary material for: Global and regional burden of chronic respiratory disease in 2016 arising from non-infectious airborne occupational exposures: a systematic analysis for the Global Burden of Disease Study 2016
Source: Occup Environ Med. 2020 Feb 13;77(3):142–50. doi: 10.1136/oemed-2019-106013 (PMC7035690; doi:10.1136/oemed-2019-106013)
Supplement: Supplementary data [file oemed-2019-106013supp001.pdf]

Global and regional burden of chronic respiratory disease in 2016 arising from non-infectious airborne occupational exposures: a systematic analysis for the Global Burden of Disease Study 2016

GBD 2016 occupational exposure to respiratory risk factors collaborators

SUPPLEMENTARY MATERIAL

APPENDIX 1: FURTHER DISCUSSION

PMGF and SHS and COPD

Using the GBD 2016 methodology, the PAFs for occupationally related COPD for 2000 were estimated to be 17% (DALYs) and 16% (deaths)[1]. Since the same set of industry-based RRs were used for all regions, the variation in PAFs between regions must come primarily from differences in the industry distribution, and because the estimates for the proportion of exposed persons within a given industry was different for LMI regions compared to high-income regions. The 41% fall in the occupationally related COPD death rate between 1990 and 2016 was considerably higher than the fall in the rate of all-cause COPD deaths (23%) over the same period[1]. Given the small variation in the PAFs over time, this suggests the variation in occupationally-related rates over time was due to a combination of changes in industry distribution and underlying changes in the prevalence of the disease in the general community.

There was uncertainty in the prevalence and level of exposure to PMGF in different industries. The prevalences used in the analysis are based on the limited published data available[2-18] and expert opinion, and it is unclear whether the effect of this uncertainty would have been likely to overestimate or to underestimate the true burden. Estimates of SHS were based on CAREX – the strengths and limitations of this approach are considered elsewhere[19].

Another issue to take into account is that the analysis of PMGF leading to COPD did not explicitly assume a latency between exposure and occurrence of symptoms, although there was an assumption of continuing risk once exposure ceased. The population at risk was based on those currently exposed, with an attempt to use this to estimate ever-exposed, without attempting to formally take turnover into account. This approach was adopted because there was considered insufficient information at the time to support the use of a different approach. However, it is likely this approach can be improved for future analyses by taking into account latency and persistence of risk.

The measures of relative risk for the COPD analysis were based on a meta-analysis conducted for this study. There were few studies that provided valid estimates of outcome (most studies based the diagnosis of COPD on self-report) and of exposure (most did not use a job-exposure matrix or equivalent), and the final relative risk estimates were based on the data from only two studies, both from the United States[14 17]. The estimates are expected to be appropriate for the exposure circumstances included in the current analysis, but there will be some uncertainty given that this is a global study and the included studies were based on a high-income country. The relationship between exposure and disease occurrence is likely to be similar across regions, but it is less clear if the exposure circumstances would be similar to those experienced by the workers included in the studies on which the relative risk estimates were based. The approach used assumed that "high" exposure and "low" exposure levels would be similar in all regions, but that the prevalence of exposure was greater in LMI regions, and generally that a higher proportion of those exposed in LMI regions were exposed at a high level.

The main determinant of exposure is likely to be the job task, and job task is likely to be related more to occupation than industry. Exposure estimates for PMGF and SHS were based on industry rather than occupation because most of the potentially useable exposure information in the literature was based on industry rather than occupation. However, the analysis was undertaken using estimates of persons exposed at high, low, or background levels, attempting to match the exposure circumstances to those present in the studies that provided the relative risk estimates. So, basing the exposure prevalence measures on industry should be a reasonable approach. Nevertheless, there is considerable scope for improvement in the assessment of the prevalence of exposure to PMGF.

Many of the control measures for SHS are likely to be different to those for PMGF. Therefore, there is interest in examining the burden arising from SHS and PMGF separately. The main relevant results are summarised here and in Table S3. Of the 460,000 deaths (382,000–551,000) from COPD, 92% were estimated to have been caused by PMGF and 11% by SHS (the percentages add to more than 100 because both exposures could contribute to the same death). Of the 10.7 million DALYs, 86% were estimated to

have been caused by PMGF and 17% by SHS. Males accounted for 76% of the PMGF-related deaths and 68% of the SHS-related deaths (the equivalent percentages for DALYs were almost exactly the same – PMGF 75%; SHS 67%).

For SHS, the use of CAREX estimates of exposure prevalence (adjusted depending on level of development in each country), which were based on results for 1990–1993 for Western Europe, has the advantage that it reflects past exposure prevalence, which appears reasonable for estimates for 2016 given the latency for exposures resulting in COPD. However, only having a single estimate means comparisons of burden across time do not take into account changes in smoking habits and thus associated changes in SHS exposure prevalence. The adjustment between regions based on level of development was also blunt. The extent of direction of any resultant bias is hard to predict, with a probable over-estimation of SHS exposure prevalence in recent years at least partially offset by a probable underestimation of SHS exposure prevalence in LMI countries. Approaches to overcome these limitations are being considered for future GBD analyses.

Asthmagens and asthma

The rates of occupational asthma deaths and DALYs reflect a combination of the PAF and the rate of all asthma in the relevant region. The all-asthma rates appear to have the major influence on this, with the six regions with the highest rates of occupationally related asthma all being the six regions with the highest overall rates of asthma deaths. The exceptionally high rate of occupational asthma deaths in Oceania (2.4 times that of the region with second-highest rate) is a direct reflection of the overall asthma death rates estimated in GBD 2016 (where the asthma death rate in Oceania was 2.6 times that of the region with the second-highest rate)[1], the cause of which is not clear. The significant fall in the rate of occupationally related asthma deaths (and DALYs) between 1990 and 2016 is heavily influenced by changes in the rate of all-cause asthma, with the rate of all-cause asthma deaths dropping by 37% over the 26-year period and the occupational exposure PAF actually increasing considerably (28%).

The analysis was based on a good-quality population-based study that was conducted in Finland[20 21]. For many occupations, the relevant exposures in Finland are likely to be similar to those in other high-income countries. For farming, where the Nordic countries' practices are likely to be importantly different from those in countries with a warmer climate, the relative risk estimate from the Finnish study was replaced with one from what was considered a good-quality study covering much of Western Europe[22]. These approaches seem appropriate for high-income countries, but they are likely to be less so for LMI countries. The high relative risk for mining raises the possibility of an overlap in the diagnosis of asthma and COPD.

As mentioned in the main paper, the CRA study estimated a PAF of 11% based on DALYs and 17% based on deaths (and estimated 38,000 deaths in 2000)[23]. The CRA approach used the same relative risks based on occupation as used in this study and a similar approach to estimating the prevalence of exposed workers. Therefore, the difference in the PAF must have arisen primarily from changes in the employment distribution and slight differences in the general methodology. Using the GBD 2016 methodology, the PAFs for occupational asthma for 2000 were estimated to be 9% for DALYs (8% for deaths)[1]. Since the relative risks were based on occupation, and the same set of relative risks was used for all regions, variation in the PAF between regions arose primarily from differences in occupation distribution and employment participation at different ages.

Pneumoconioses

The estimate of 21,500 deaths is considerably lower than the estimate of about 260,000 deaths in GBD 2013[24]. The difference arose primarily from a change in the approach used to allocate heart failure deaths to a specific cause of death category, with the GBD 2013 approach likely to have led to a major overestimation. The current estimate is also moderately lower than the estimate of 36,000 in GBD 2015[25], with the differences due to improvements in estimates of causes of death. The current estimate is considered more accurate than previous estimates, but nevertheless remains subject to uncertainty arising from inaccurate cause of death coding in the source data, which is considered likely to have led to an

underestimate, rather than an overestimate, of the burden arising from exposure to pneumoconiotic dusts. In addition, pneumoconiosis predisposes to tuberculosis, meaning the true burden of disease related to pneumoconiosis is likely to be higher than reported in the current analysis[26].

The CRA study estimated 30,000 deaths from the three main pneumoconioses (silicosis, asbestosis, and coal workers' pneumoconiosis) and 36,000 from all pneumoconioses[23]. Very different approaches were used in the two analyses. The CRA 2000 estimates were made using estimates of absolute risk and exposure and had more scope for error. The GBD 2016 estimates were based on sophisticated modelling of available information on incidence, prevalence, and mortality and are considered more accurate, with the proviso about the "Other pneumoconioses" category mentioned in the main paper.

Other methodological considerations

The main general uncertainties have been considered in detail in the companion overview paper[27]. Methodological issues specific to the three main outcomes of interest have already been considered above in the relevant sections. General issues of particular relevance to the presented analysis are considered here.

Industry (for PMGF and SHS) and occupation (for asthmagens) were used as the basis for the risk factor exposure prevalence measures, which in turn were used to produce the PAF estimates. Since the relative risks were constant over time, changes in the PAFs over time are primarily due to differences over time in terms of occupation and industry distribution and employment participation at different ages, rather than to measured changes in the exposures for a given occupation or industry. Ideally, direct measures of the relevant hazards would be used. For COPD and asthma, the relevant exposures to measure are often hard to characterise, and they are hard to measure even if they can be characterised, which means the data on absolute or cumulative exposure are not available for the vast majority of relevant exposures. Also, the risk measures in the literature are typically available on the basis of occupation or industry, or on the basis of high exposure or low exposure. Therefore, using industry (for PMGF and SHS) and occupation (for

asthmagens) was the only feasible approach that could be used to assign exposure prevalence. However, it may be possible to use broad measures of exposure to adjust for changes in exposure over time, and the development of job-exposure matrices suitable for use in occupational COPD[28] and asthma[29] studies raises the prospect of being able to incorporate more direct measures of relevant exposures in future analyses.

The relative risk estimates used were those considered the most appropriate available, but it is likely that there have been mismatches between the relative risk estimates used and the exposure circumstances to which they have been applied for some workers, countries, and time periods. This is because the relative risk estimates came from high-income countries and covered a range of time periods, the included workers had varying length and intensity of exposure, and the time since first exposure varied. This could have resulted in over- or underestimation of the relevant and relative burden for particular regions. The potential misclassification of exposure, and the "healthy worker effect" in the source studies would both be expected to have led to some underestimation of the true effect of the exposures (as reflected in the relative risks) and thus to an underestimation of the burden.

Tobacco smoking is an important modifier of the effects of many of the relevant exposures and of course is the most important cause of COPD. Therefore, changes over time and differences between regions will have been influenced by trends over time and regional differences in the prevalence of tobacco smoking in addition to the factors mentioned earlier. Similarly, no account is taken of environmental exposures away from work. The study does not include a range of potentially important occupational respiratory diseases, such as respiratory infections[30], other occupational causes of fibrosis apart from pneumoconioses and lung disease arising from nanoparticle exposure[31].

Table S1 PGMF exposure proportion by level of exposure, economic activity, and country income level, 2015[1]

Economic activity	High income		Low & middle income	
	High[2]	Low[2]	High	Low
Agriculture, hunting, forestry, and fishing	0.10	0.50	0.10	0.70
Mining and quarrying	0.10	0.70	0.40	0.40
Manufacturing	0.10	0.70	0.40	0.40
Electricity, gas, and water	0.10	0.50	0.10	0.70
Construction	0.10	0.70	0.40	0.40
Wholesale and retail trade and restaurants and hotels	0	0.05	0	0.10
Transport, storage, and communication	0.10	0.50	0.10	0.70
Financing, insurance, real estate, and business services	0	0.05	0	0.10
Community, social, and personal services	0	0.05	0	0.10

1: PMGF=Particulate matter, gases and fumes

2: "High" and "low" exposure: these are qualitative categories judged to most closely match the exposures in the studies that provided the risk measures.

Table S2 Relative risks and 95% uncertainty intervals for asthma, by occupation and sex

	Male			Female		
**Occupation**	**RR[1]**	**95% LL[2]**	**95% UL[3]**	**RR**	**95% LL**	**95% UL**
Background	1			1		
Administration	1			1		
Technical	1.05	0.98	1.12	1.06	1.03	1.10
Sales	1.14	1.05	1.23	1.13	1.08	1.18
Agriculture	1.50	1.11	2.03	1.50	1.11	2.03
Mining	1.95	1.58	2.40	1.95	1.58	2.40
Transport	1.31	1.22	1.40	1.22	1.13	1.31
Manufacturing	1.56	1.47	1.65	1.33	1.27	1.39
Services	1.53	1.42	1.66	1.41	1.35	1.46

1: RR=Relative risk
2: 95% uncertainty interval lower limit
3: 95% uncertainty interval upper limit
Based on Karjalainen and Nurminen, 2001, 2002[20 21]

Table S3 Global occupational-attributable deaths and DALYs from chronic obstructive pulmonary disease due to airborne exposures by risk factor and sex, 2016 (number, percent, and proportion [95% uncertainty interval])[1]

	Deaths				DALYs			
**Risk factor**	**Males[2]**	**Females**	**Total**	**%[3]**	**Males**	**Females**	**Total**	**%[4]**
PMGF[5]	321,113 (250,173 - 397,847)	103,133 (73,902 - 136,929)	424,266 (349,978 - 507,545)	92.2	6,971,890 5,650,651 – 8,303,288)	2,182,656 (1,704,845 – 2,688,233)	9,154,546 (7,771,090 – 10,539,367)	85.7
SHS[6]	35,161 (16,188 – 63,160)	16,741 (7,687 – 30,049)	51,903 (23,787 – 91,431)	11.3	1,226,294 (558,253 – 2,229,256)	593,698 (278,653 – 1,050,058)	1,819,991 831,200- 3,260,920)	17.0
**Total**	**343,122** (270,900 - 422,000)	**116,958** (87,100 - 153,300)	**460,080** (381,500 - 551,300)	**100.0**	**7,969,986** (6,518,000 - 9,469,200)	**2,717,967** (2,143,400 - 3,328600)	**10,687,953** (9,019,900 - 12,517,000)	**100.0**

1: DALY=Disability-adjusted life year.
2: The numbers in brackets in the whole table are 95% uncertainty intervals
3: Percentage of deaths from chronic obstructive pulmonary disease (COPD) that were due to this risk factor. The individual percentages don't add to 100 because both exposures could contribute to a single death.
4: Percentage of DALYs from COPD that were due to this risk factor. The individual percentages don't add to 100 because both exposures could contribute to a single event.
5: Particulate matter, gases and fumes causing COPD
6: Second-hand smoke causing COPD

Figure S1 Occupation-attributable deaths from chronic respiratory disease due to exposure to PMGF and SHS, 2016

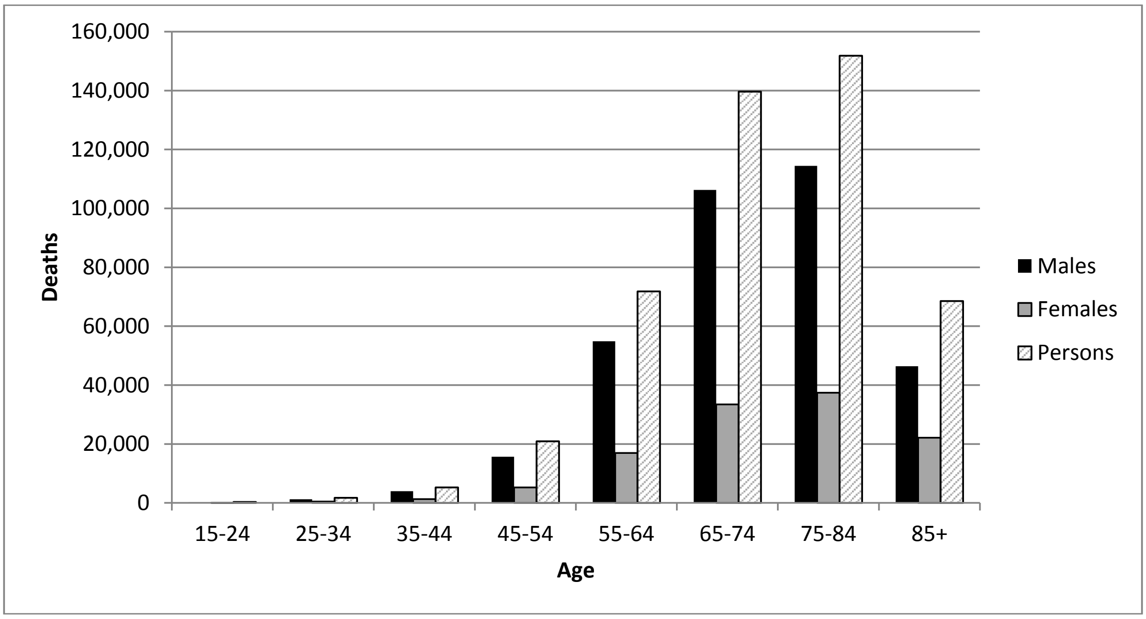

PMGF= Particulate matter, gases and fumes; SHS=Second-hand smoke.

Figure S2 Occupation-attributable deaths from chronic respiratory disease due to exposure to PMGF and SHS, 2016 (per 100,000 persons)

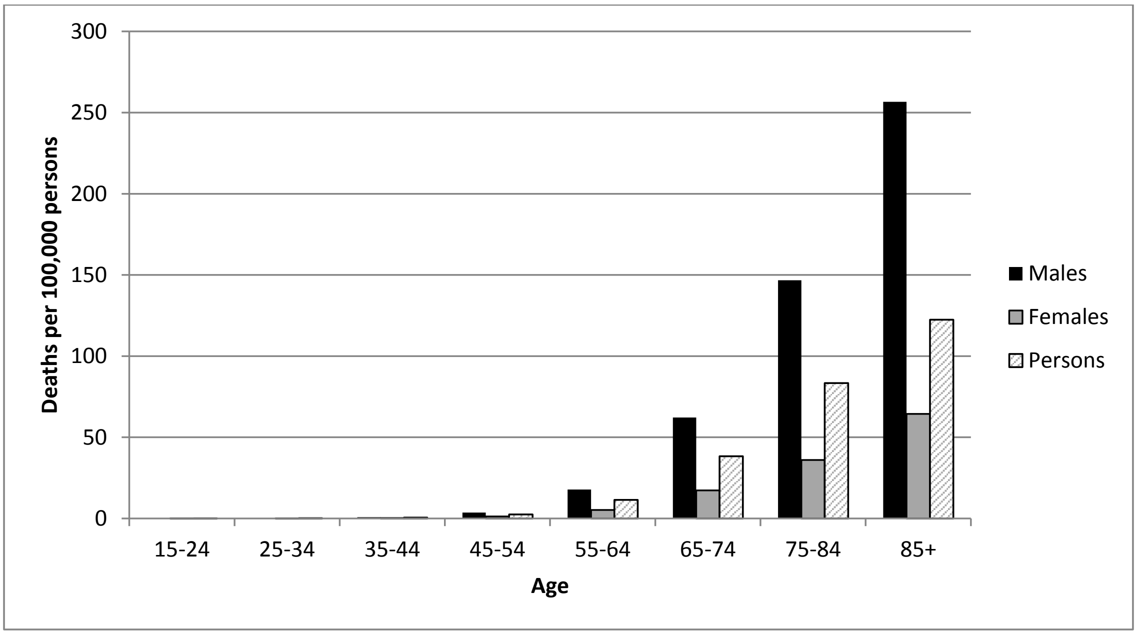

PMGF= Particulate matter, gases and fumes; SHS=Second-hand smoke.

Figure S3 Occupation-attributable DALYs from chronic respiratory disease due to exposure to PMGF and SHS, 2016

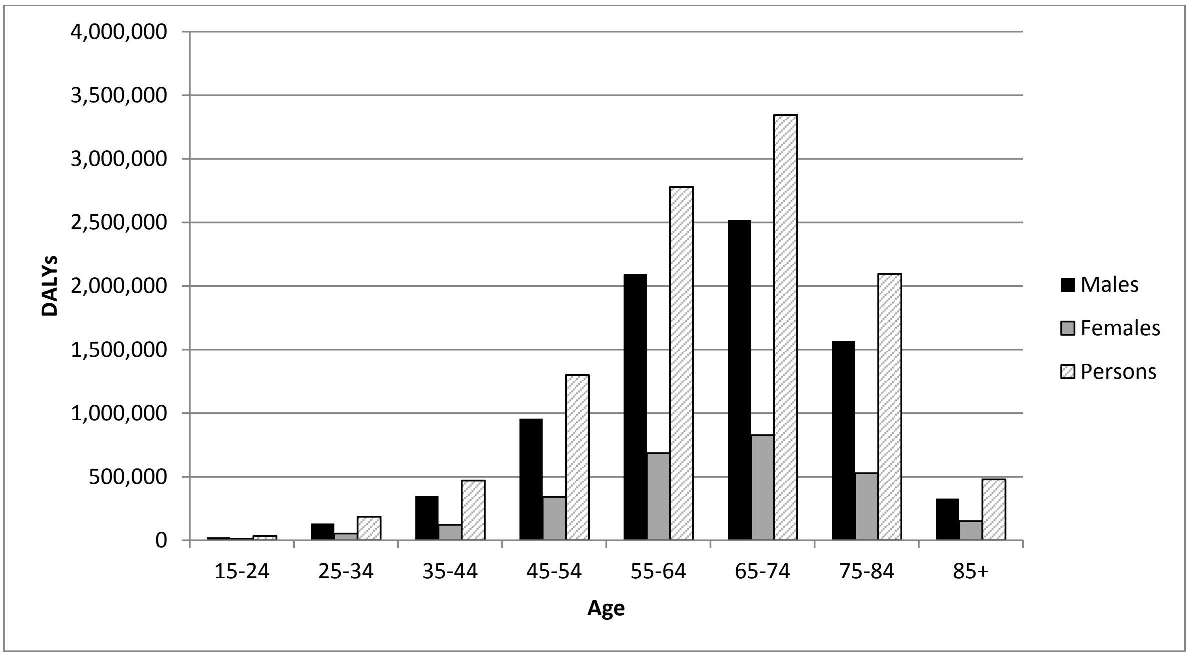

DALY=Disability-adjusted life year; PMGF= Particulate matter, gases and fumes; SHS=Second-hand smoke.

Figure S4 Occupation-attributable DALYs from chronic respiratory disease due to exposure to PMGF and SHS, 2016 (per 100,000 persons)

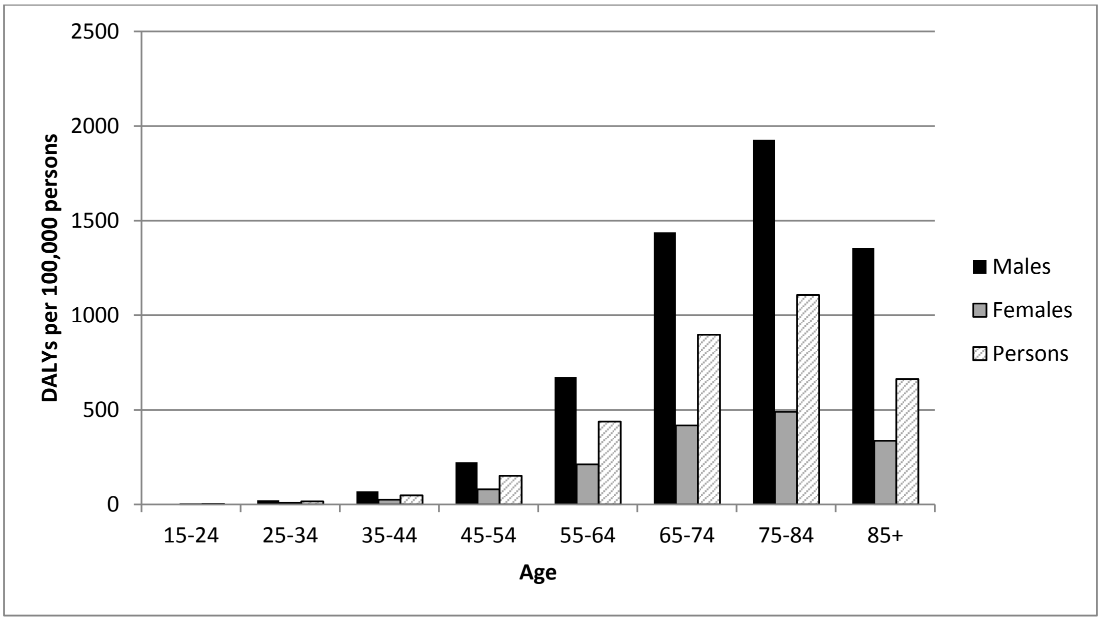

DALY=Disability-adjusted life year; PMGF= Particulate matter, gases and fumes; SHS=Second-hand smoke.

Figure S5 Occupation-attributable deaths due to exposure to asthmagens, 2016

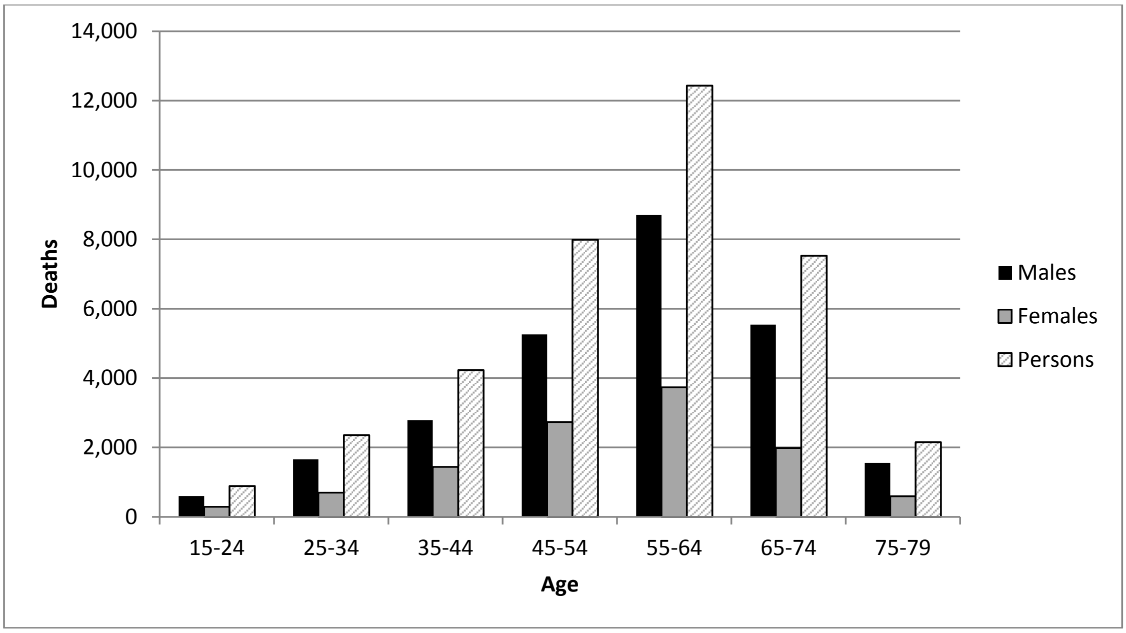

Figure S6 Occupation-attributable deaths due to exposure to asthmagens, 2016 (per 100,000 persons)

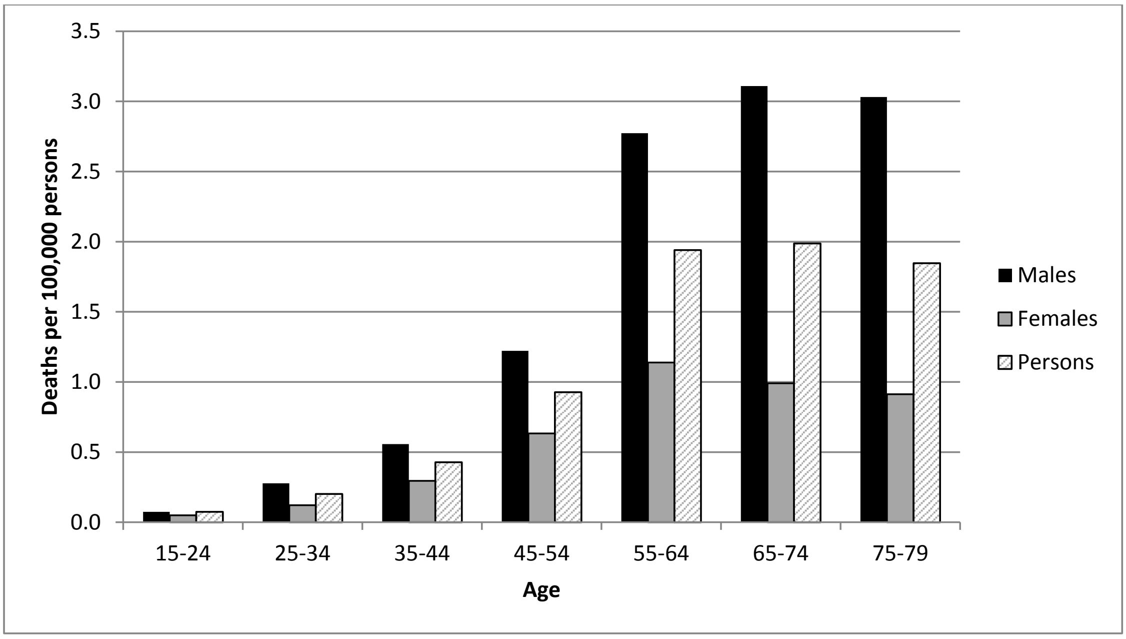

Figure S7 Occupation-attributable DALYs due to exposure to asthmagens, 2016

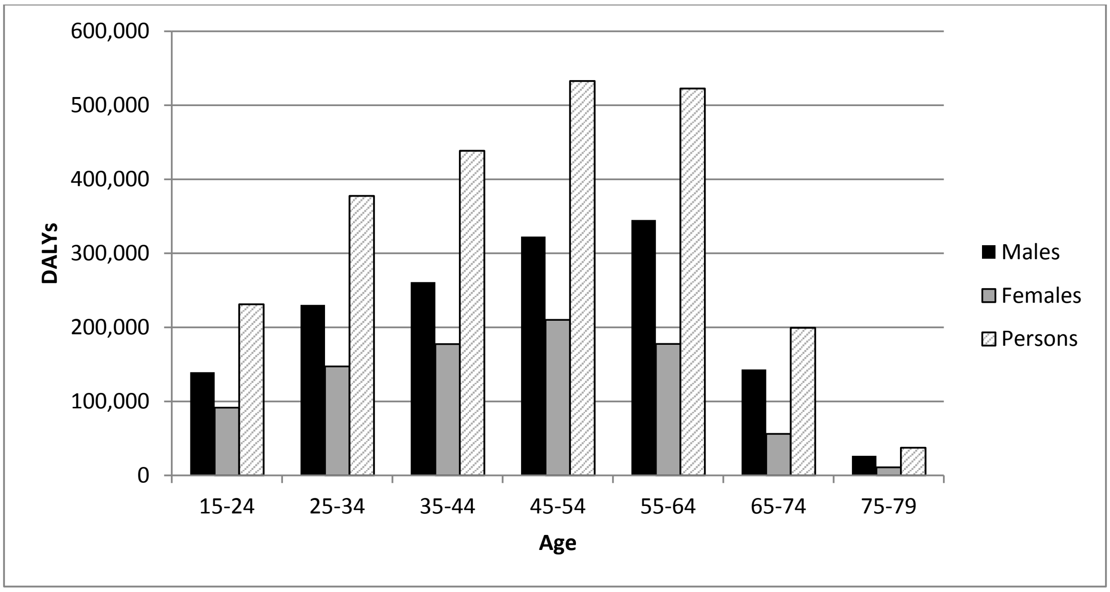

DALY=Disability-adjusted life year.

Figure S8 Occupation-attributable DALYs due to exposure to asthmagens, 2016 (per 100,000 persons)

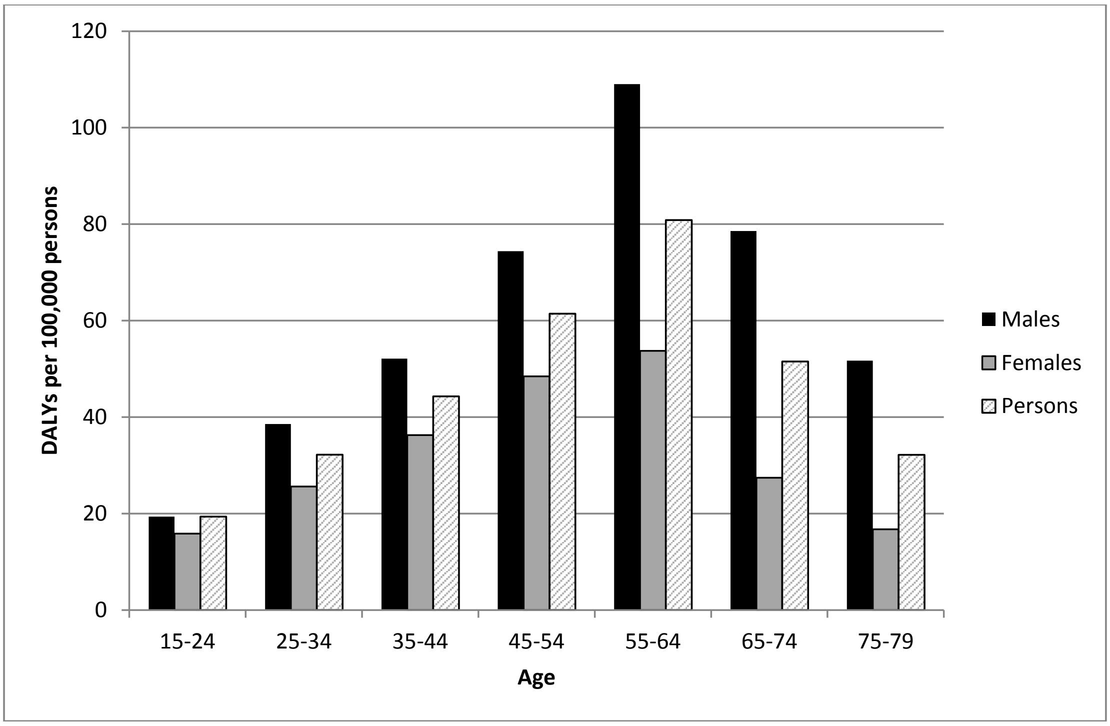

DALY=Disability-adjusted life year.

Figure S9 Occupation-attributable deaths due to exposure to pneumoconiotic dusts, 2016

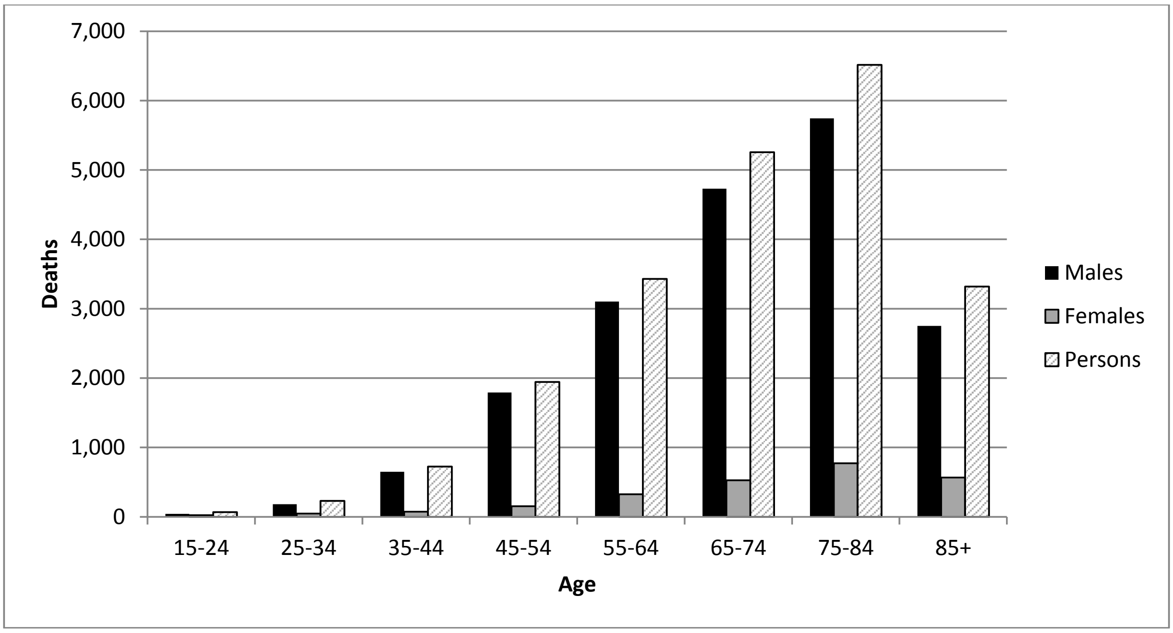

Figure S10 Occupation-attributable deaths due to exposure to pneumoconiotic dusts, 2016 (per 100,000 persons)

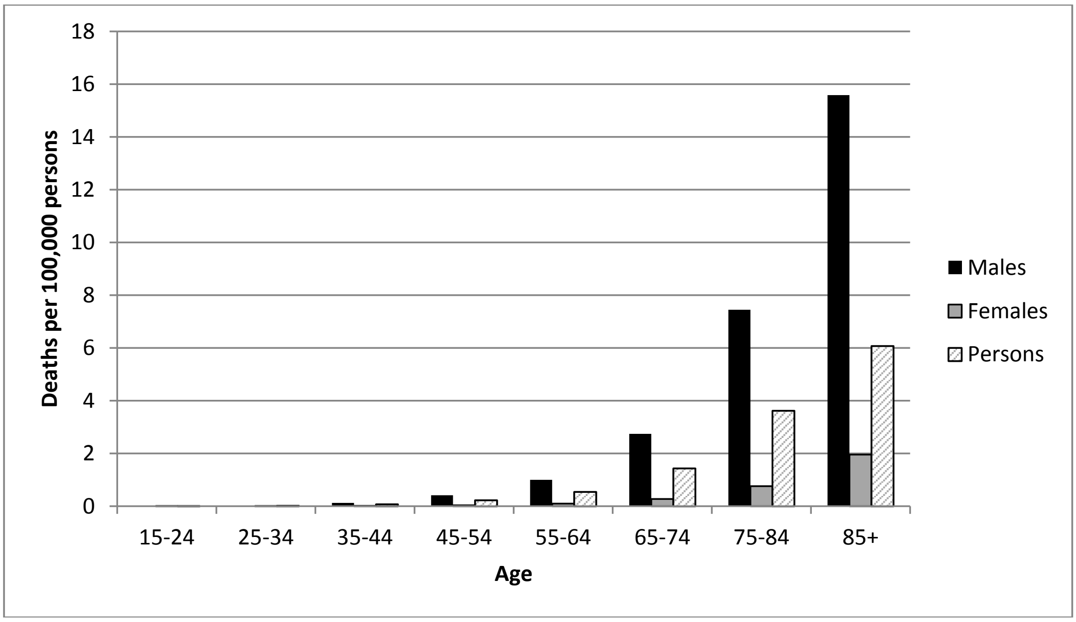

Figure S11 Occupation-attributable DALYs due to exposure to pneumoconiotic dusts, 2016

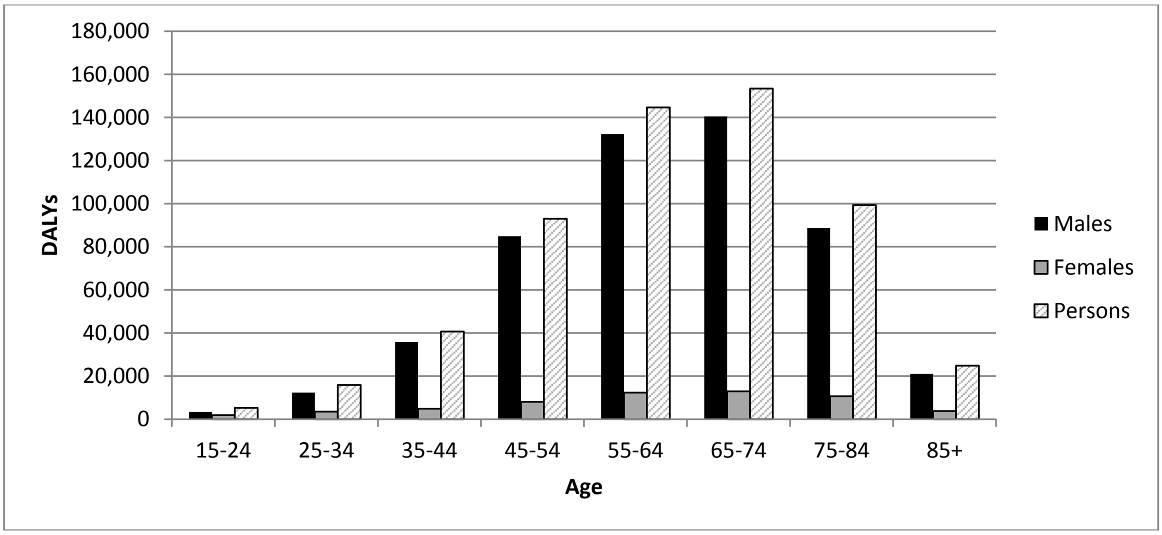

DALY=Disability-adjusted life year.

Figure S12 Occupation-attributable DALYs due to exposure to pneumoconiotic dusts, 2016 (per 100,000 persons)

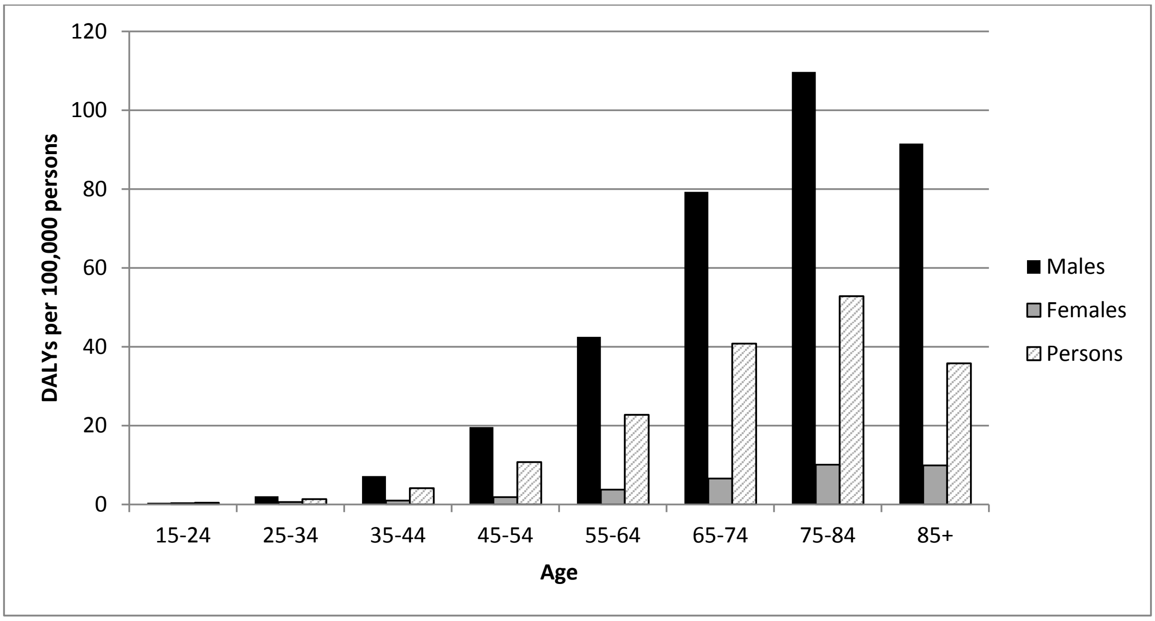

DALY=Disability-adjusted life year.

REFERENCES

1. Institute for Health Metrics and Evaluation. GBD Compare. 2017 ed. Seattle, WA: IHME, University of Washington, 2017.
2. Jindal S. Silicosis in India: past and present. *Current Opinion in Pulmonary Medicine* 2013;19(2):163-68.
3. Carey R, Driscoll T, Peters S, et al. Estimated prevalence of exposure to occupational carcinogens in Australia (2011-2012). *Occupational and Environmental Medicine* 2014;71(1):55-62.
4. Spankie S, Cherrie JW. Exposure to grain dust in Great Britain. *The Annals of occupational hygiene* 2012;56(1):25-36. doi: 10.1093/annhyg/mer084 [published Online First: 2011/10/07]
5. Schoonover T, Conroy L, Lacey S, et al. Personal exposure to metal fume, NO2, and O3 among production welders and non-welders. *Ind Health* 2011;49(1):63-72. [published Online First: 2010/09/09]
6. Fayerweather WE, Trumbore DC, Johnson KA, et al. Quantitative exposure matrix for asphalt fume, total particulate matter, and respirable crystalline silica among roofing and asphalt manufacturing workers. *Inhalation toxicology* 2011;23(11):668-79. doi: 10.3109/08958378.2011.604107 [published Online First: 2011/09/02]
7. Swanepoel AJ, Rees D, Renton K, et al. Quartz exposure in agriculture: literature review and South African survey. *The Annals of occupational hygiene* 2010;54(3):281-92. doi: 10.1093/annhyg/meq003 [published Online First: 2010/02/23]
8. Anttila P, Heikkila P, Makela M, et al. Retrospective exposure assessment for carcinogenic agents in bitumen waterproofing industry in Finland and denmark. *The Annals of occupational hygiene* 2009;53(2):139-51. doi: 10.1093/annhyg/men082 [published Online First: 2009/02/05]
9. Williams PR, Phelka AD, Paustenbach DJ. A review of historical exposures to asbestos among skilled craftsmen (1940-2006). *Journal of toxicology and environmental health Part B, Critical reviews* 2007;10(5):319-77. doi: 10.1080/10937400601034191 [published Online First: 2007/08/10]
10. Rushton L. Chronic obstructive pulmonary disease and occupational exposure to silica. *Rev Environ Health* 2007;22(4):255-72. [published Online First: 2008/03/21]
11. Schenker M. Exposures and health effects from inorganic agricultural dusts. *Environ Health Perspect* 2000;108 Suppl 4:661-4. [published Online First: 2000/08/10]
12. Ellen HH, Bottcher RW, von Wachenfelt E, et al. Dust levels and control methods in poultry houses. *Journal of agricultural safety and health* 2000;6(4):275-82. [published Online First: 2001/02/24]
13. Dost AA, Redman D, Cox G. Exposure to rubber fume and rubber process dust in the general rubber goods, tyre manufacturing and retread industries. *The Annals of occupational hygiene* 2000;44(5):329-42. [published Online First: 2000/08/10]
14. Blanc P, Iribarren C, Trupin L, et al. Occupational exposures and the risk of COPD: dusty trades revisited. *Thorax* 2009;64(1):6-12.
15. Blanc PD, Eisner MD, Earnest G, et al. Further exploration of the links between occupational exposure and chronic obstructive pulmonary disease. *J Occup Environ Med* 2009;51(7):804-10. doi: 10.1097/JOM.0b013e3181a7dd4e [published Online First: 2009/06/17]
16. Trupin L, Earnest G, San Pedro M, et al. The occupational burden of chronic obstructive pulmonary disease. *The European respiratory journal* 2003;22(3):462-9. [published Online First: 2003/10/01]
17. Weinmann S, Vollmer W, Breen V, et al. COPD and occupational exposures: a case-control study. *Journal of Occupational and Environmental Medicine* 2008;50(5):561-69.
18. Zock JP, Sunyer J, Kogevinas M, et al. Occupation, chronic bronchitis, and lung function in young adults. An international study. *Am J Respir Crit Care Med* 2001;163(7):1572-7. doi: 10.1164/ajrccm.163.7.2004195 [published Online First: 2001/06/13]
19. GBD 2016 Occupational Carcinogens Collaborators. Global and regional burden of cancer in 2016 arising from occupational exposure to carcinogens: a systematic analysis for the Global Burden of Disease Study 2016. *Occupational and Environmental Medicine* 2019:Submitted.
20. Karjalainen A, Kurppa K, Martikanen R, et al. Exploration of asthma risk by occupation -extended analysis of an incidence study of the Finnish population. *Scandinavian Journal of Work, Environment and Health* 2002;28:49–57.
21. Karjalainen A, Kurppa K, Martikanen R, et al. Work is related to a substantial portion of adult-onset asthma incidence in the Finnish population. *American Journal of Respiratory and Critical Care Medicine* 2001;164:565–68.

22. Kogevinas M, Anto J, Sunyer J, et al. Occupational asthma in Europe. *Lancet* 1999;353:1750–54.
23. Driscoll T, Nelson D, Steenland K, et al. The global burden of non-malignant respiratory disease due to occupational airborne exposures. *American Journal of Industrial Medicine* 2005;48:432-45.
24. GBD 2013 Mortality and Causes of Death Collaborators, Naghavi M, Wang H, et al. Global, regional, and national age-sex specific all-cause and cause-specific mortality for 240 causes of death, 1990-2013: a systematic analysis for the Global Burden of Disease Study 2013. *Lancet* 2015;385(9963):117-71.
25. GBD 2015 Risk Factors Collaborators. Global, regional, and national comparative risk assessment of 79 behavioural, environmental and occupational, and metabolic risks or clusters of risks, 1990-2015: a systematic analysis for the Global Burden of Disease Study 2015. *Lancet* 2016;388(10053):1659-724. doi: 10.1016/S0140-6736(16)31679-8
26. Rees D, Murray J. Silica, silicosis and tuberculosis. *The international journal of tuberculosis and lung disease : the official journal of the International Union against Tuberculosis and Lung Disease* 2007;11(5):474-84. [published Online First: 2007/04/19]
27. GBD 2016 Occupational Risk Factors Collaborators. Global and regional burden of disease and injury arising from occupational exposures in 2016: a systematic analysis for the Global Burden of Disease Study 2016. *Occupational and Environmental Medicine* 2019:Submitted.
28. Matheson MC, Benke G, Raven J, et al. Biological dust exposure in the workplace is a risk factor for chronic obstructive pulmonary disease. *Thorax* 2005;60(8):645-51. doi: 10.1136/thx.2004.035170 [published Online First: 2005/08/03]
29. Kennedy SM, Le Moual N, Choudat D, et al. Development of an asthma specific job exposure matrix and its application in the epidemiological study of genetics and environment in asthma (EGEA). *Occup Environ Med* 2000;57(9):635-41. [published Online First: 2000/08/10]
30. Baussano I, Nunn P, Williams B, et al. Tuberculosis among health care workers. *Emerging infectious diseases* 2011;17(3):488-94. doi: 10.3201/eid1703.100947 [published Online First: 2011/03/12]
31. Traboulsi H, Guerrina N, Iu M, et al. Inhaled Pollutants: The Molecular Scene behind Respiratory and Systemic Diseases Associated with Ultrafine Particulate Matter. *International journal of molecular sciences* 2017;18(2) doi: 10.3390/ijms18020243 [published Online First: 2017/01/27]
