# Supplementary material for: Global and regional burden of chronic respiratory disease in 2016 arising from non-infectious airborne occupational exposures: a systematic analysis for the Global Burden of Disease Study 2016
Source: Occup Environ Med. 2020 Feb 13;77(3):142–50. doi: 10.1136/oemed-2019-106013 (PMC7035690; doi:10.1136/oemed-2019-106013)
Supplement: Supplementary data [file oemed-2019-106013supp002.pdf]

GBD 2016 Respiratory Exposures Collaborators

Tim Driscoll[1], Kyle Steenland[2], Neil Pearce[3], Lesley Rushton[4], Sally J. Hutchings[5], Kurt Straif[6], Degu Abate[7], Dilaram Acharya[8,9], Anurag Agrawal[10,11], Fares Alahdab[12], Kefyalew Addis Alene[13,14], Sofia Androudi[15], Mina Anjomshoa[16], Carl Abelardo T. Antonio[17,18], Olatunde Aremu[19], Zerihun Ataro[7], Alaa Badawi[20,21], Joseph Adel Mattar Banoub[22], Suzanne Lyn Barker-Collo[23], Neeraj Bedi[24,25], Derrick A. Bennett[26], Robert Bernstein[214] ,Mircea Beuran[27,28], Krittika Bhattacharyya[29,30], Ali Bijani[31], Zahid A. Butt[32,33], Juan J. Carrero[34], Carlos A. Castañeda-Orjuela[35,36], Odgerel Chimed-Ochir[37], Lalit Dandona[38,39], Rakhi Dandona[38,39], Anh Kim Dang[40], Ahmad Daryani[41], Beruk Berhanu Desalegn[42], Samath Dhamminda Dharmaratne[39,43], Shirin Djalalinia[44], Eleonora Dubljanin[45], Soheil Ebrahimpour[46], Ziad El-Khatib[47], Mohammad Fareed[48], Andre Faro[49], Eduarda Fernandes[50], Florian Fischer[51], Takeshi Fukumoto[52,53], Silvano Gallus[54], Teklu Gebrehiwo Gebremichael[55], Kebede Embaye Gezae[56], Ayman Grada[57], Yuming Guo[58,59], Rahul Gupta[60,61], Arvin Haj-Mirzaian[62,63], Arya Haj-Mirzaian[62,64], Samer Hamidi[65], Mehedi Hasan[66], Milad Hasankhani[67], Simon I. Hay[39,68], Chi Linh Hoang[69], Michael K. Hole[70], H Dean Hosgood[71], Mihaela Hostiuc[72,73], Sorin Hostiuc[74,75], Seyed Sina Naghibi Irvani[76,77], Sheikh Mohammed Shariful Islam[78,79], Mihajlo Jakovljevic[80], Ravi Prakash Jha[81], Jost B. Jonas[82,83], Amaha Kahsay[84], Amir Kasaeian[85,86], Norito Kawakami[87], Yousef Saleh Khader[88], Morteza Abdullatif Khafaie[89], Ejaz Ahmad Khan[90], Mohammad Hossein Khosravi[91], Jagdish Khubchandani[92], Yun Jin Kim[93], Ruth W. Kimokoti[94], Adnan Kisa[95,96], Manolis Kogevinas[215], Soewarta Kosen[97], Parvaiz A. Koul[98], Ai Koyanagi[99,100], Barthelemy Kuate Defo[101,102], G Anil Kumar[38], Dharmesh Kumar Lal[38], Arman Latifi[103], James Leigh[104], Miriam Levi[105,106], Shanshan Li[58], Shai Linn[107], Narayan Bahadur Mahotra[108], Marek Majdan[109], Reza Malekzadeh[110,111], Mohammad Ali Mansournia[112], Francisco Rogerlândio Martins-Melo[113], Benjamin Ballard Massenburg[114], Varshil Mehta[115], Addisu Melese[116], Mulugeta Melku[13], Ziad A. Memish[117,118], Walter Mendoza[119], Tuomo J. Meretoja[120], Tomislav Mestrovic[121,122], GK Mini[123,124], Erkin M. Mirrakhimov[125,126], Babak Moazen[127,128], Naser Mohammad Gholi Mezerji[129], Shafiu Mohammed[127,130], Ali H Mokdad[39,68], Lorenzo Monasta[131], Yoshan Moodley[132], Mahmood Moosazadeh[133], Ghobad Moradi[134,135], Lidia Morawska[136], Shane Douglas Morrison[137], Seyyed Meysam Mousavi[138], Ghulam Mustafa[139,140], Vinay Nangia[141], Ionut Negoi[27,28], Ruxandra Irina Negoi[142,143], Cuong Tat Nguyen[40], Trang Huyen Nguyen[69], Molly R. Nixon[39], Richard Ofori-Asenso[144], Felix Akpojene Ogbo[145], Andrew T. Olagunju[146,147], Bolajoko Olubukunola Olusanya[148], Mahesh P A[149], Songhomitra Panda-Jonas[150], Eun-Kee Park[151], Sanghamitra Pati[152], Mostafa Qorbani[153], Anwar Rafay[154,155], Alireza Rafiei[156,157], Fakher Rahim[158,159], Vafa Rahimi-Movaghar[160], Fatemeh Rajati[161], Robert C. Reiner[39,68], Satar Rezaei[162], Leonardo Roever[163], Luca Ronfani[131], Gholamreza Roshandel[110,164], Basema Saddik[165], Saeid Safiri[166], Mohammad Ali Sahraian[167], Abdallah M. Samy[168], David C. Schwebel[169], Sadaf G. Sepanlou[110,111], Berrin Serdar[170,171], Masood Ali Shaikh[172], Aziz Sheikh[173,174], Mika Shigematsu[175], Rahman Shiri[176], Reza Shirkoohi[177,178], Si Si[58], João Pedro Silva[179], Dhirendra Narain Sinha[180], Moslem Soofi[181], Joan B. Soriano[182,183], Chandrashekhar T. Sreeramareddy[184], Jeffrey D. Stanaway[39,68], Mark A. Stokes[185], Mu'awiyyah Babale Sufiyan[186], Ipsita Sutradhar[66], Rafael Tabarés-Seisdedos[187,188], Ken Takahashi[104], Yonatal Mesfin Tefera[189,190], Mohamad-Hani Temsah[118,191], Marcos Roberto Tovani-Palone[192], Bach Xuan Tran[193], Khanh Bao Tran[194,195], Lorainne Tudor Car[196], Irfan Ullah[197,198], Pascual R. Valdez[199,200], Job F. M. van Boven[201,202], Tommi Juhani Vasankari[203], Francesco S. Violante[204,205], Giang Thu Vu[69], Gregory R. Wagner[206], Yasir Waheed[207], Yuan-Pang Wang[208], Biruck Desalegn Yirsaw[209], Naohiro Yonemoto[210], Chuanhua Yu[211,212], Mohammad Zamani[213], and Stephen S. Lim[39,68].

Affiliations

1 Sydney School of Public Health, University of Sydney, Sydney, NSW, Australia.
2 Rollins School of Public Health, Emory University, Atlanta, GA, USA.
3 Department of Medical Statistics, London School of Hygiene & Tropical Medicine, London, UK.
4 Department of Epidemiology and Biostatistics, Imperial College London, London, UK.
5 School of Health Sciences, University of Manchester, Manchester, UK.
6 Section of Evidence Synthesis and Classification, International Agency for Research on Cancer, Lyon, France.
7 Department of Medical Laboratory Sciences, Haramaya University, Harar, Ethiopia.
8 Department of Preventive Medicine, Dongguk University, Gyeongju, South Korea.
9 Department of Community Medicine, Kathmandu University, Devdaha, Nepal.
10 Research Area for Informatics and Big Data, CSIR Institute of Genomics and Integrative Biology, Delhi, India.
11 Department of Internal Medicine, Baylor College of Medicine, Houston, TX, USA.
12 Evidence Based Practice Center, Mayo Clinic Foundation for Medical Education and Research, Rochester, MN, USA.
13 Institute of Public Health, University of Gondar, Gondar, Ethiopia.
14 Research School of Population Health, Australian National University, Canberra, ACT, Australia.
15 Department of Medicine, University of Thessaly, Volos, Greece.
16 Social Determinants of Health Research Center, Rafsanjan University of Medical Sciences, Rafsanjan, Iran.
17 Department of Health Policy and Administration, University of the Philippines Manila, Manila, Philippines.
18 Department of Applied Social Sciences, Hong Kong Polytechnic University, Hong Kong, China.
19 School of Health Sciences, Birmingham City University, Birmingham, UK.
20 Public Health Risk Sciences Division, Public Health Agency of Canada, Toronto, ON, Canada.
21 Department of Nutritional Sciences, University of Toronto, Toronto, ON, Canada.
22 Faculty of Medicine, Alexandria University, Alexandria, Egypt.
23 School of Psychology, University of Auckland, Auckland, New Zealand.
24 Department of Community Medicine, Gandhi Medical College Bhopal, Bhopal, India.
25 Jazan University, Jazan, Saudi Arabia.
26 Nuffield Department of Population Health, University of Oxford, Oxford, UK.
27 Emergency Hospital of Bucharest, Carol Davila University of Medicine and Pharmacy, Bucharest, Romania.
28 General Surgery Department, Carol Davila University of Medicine and Pharmacy, Bucharest, Romania.
29 Department of Statistical and Computational Genomics, National Institute of Biomedical Genomics, Kalyani, India.
30 Department of Statistics, University of Calcutta, Kolkata, India.
31 Social Determinants of Health Research Center, Babol University of Medical Sciences, Babol, Iran.
32 School of Population and Public Health, University of British Columbia, Vancouver, BC, Canada.
33 Al Shifa School of Public Health, Al Shifa Trust Eye Hospital, Rawalpindi, Pakistan.
34 Department of Medical Epidemiology and Biostatistics, Karolinska Institutet, Stockholm, Sweden.

35 Colombian National Health Observatory, National Institute of Health, Bogota, Colombia.
36 Epidemiology and Public Health Evaluation Group, National University of Colombia, Bogota, Colombia.
37 Institute of Industrial Ecological Science, University of Occupational and Environmental Health, Kitakyushu, Japan.
38 Public Health Foundation of India, Gurugram, India.
39 Institute for Health Metrics and Evaluation, University of Washington, Seattle, WA, USA.
40 Institute for Global Health Innovations, Duy Tan University, Hanoi, Vietnam.
41 Toxoplasmosis Research Center, Mazandaran University of Medical Sciences, Sari, Iran.
42 School of Nutrition, Food Science and Technology, Hawassa University, Hawassa, Ethiopia.
43 Department of Community Medicine, University of Peradeniya, Peradeniya, Sri Lanka.
44 Deputy of Research and Technology, Ministry of Health and Medical Education, Tehran, Iran.
45 Faculty of Medicine, University of Belgrade, Belgrade, Serbia.
46 Center for Infectious Diseases Research, Babol, Iran.
47 Department of Public Health Sciences, Karolinska Institutet, Stockholm, Sweden.
48 College of Medicine, Imam Muhammad Ibn Saud Islamic University, Riyadh, Saudi Arabia.
49 Department of Psychology, Federal University of Sergipe, Sao Cristovao, Brazil.
50 REQUIMTE/LAQV, University of Porto, Porto, Portugal.
51 Department of Public Health Medicine, Bielefeld University, Bielefeld, Germany.
52 Gene Expression & Regulation Program, Cancer Institute (W.I.A.), Philadelphia, PA, USA.
53 Department of Dermatology, Kobe University, Kobe, Japan.
54 Department of Environmental Health Science, Mario Negri Institute for Pharmacological Research, Milan, Italy.
55 School of Pharmacy, Mekelle University, Mekelle, Ethiopia.
56 Department of Biostatistics, Mekelle University, Mekelle, Ethiopia.
57 School of Medicine, Boston University, Boston, MA, USA.
58 School of Public Health and Preventive Medicine, Monash University, Melbourne, VIC, Australia.
59 Department of Epidemiology and Biostatistics, College of Public Health, Zhengzhou University, Zhengzhou, China.
60 March of Dimes, Arlington, VA, USA.
61 School of Public Health, West Virginia University, Morgantown, WV, USA.
62 Department of Pharmacology, Tehran University of Medical Sciences, Tehran, Iran.
63 Obesity Research Center, Research Institute for Endocrine Sciences, Shahid Beheshti University of Medical Sciences, Tehran, Iran.
64 Department of Radiology, Johns Hopkins University, Baltimore, MD, USA.
65 School of Health and Environmental Studies, Hamdan Bin Mohammed Smart University, Dubai, United Arab Emirates.
66 James P Grant School of Public Health, BRAC University, Dhaka, Bangladesh.
67 School of Nutrition and Food Sciences, Tabriz University of Medical Sciences, Tabriz, Iran.
68 Department of Health Metrics Sciences, School of Medicine, University of Washington, Seattle, WA, USA.
69 Center of Excellence in Behavioral Medicine, Nguyen Tat Thanh University, Ho Chi Minh, Vietnam.
70 Department of Pediatrics, Dell Medical School, University of Texas Austin, Austin, TX, USA.
71 Department of Epidemiology and Population Health, Albert Einstein College of Medicine, Bronx, NY, USA.

72 Department of General Surgery, Carol Davila University of Medicine and Pharmacy, Bucharest, Romania.
73 Department of Internal Medicine, Bucharest Emergency Hospital, Bucharest, Romania.
74 Faculty of Dentistry, Department of Legal Medicine and Bioethics, Carol Davila University of Medicine and Pharmacy, Bucharest, Romania.
75 Clinical Legal Medicine, National Institute of Legal Medicine Mina Minovici, Bucharest, Romania.
76 Research Institute for Endocrine Sciences, Shahid Beheshti University of Medical Sciences, Tehran, Iran.
77 Non-communicable Diseases Research Center, Tehran University of Medical Sciences, Tehran, Iran.
78 Institute for Physical Activity and Nutrition, Deakin University, Burwood, VIC, Australia.
79 Sydney Medical School, University of Sydney, Sydney, NSW, Australia.
80 Department of Global Health, Economics and Policy, Faculty of Medical Sciences, University of Kragujevac, Kragujevac, Serbia.
81 Department of Community Medicine, Banaras Hindu University, Varanasi, India.
82 Department of Ophthalmology, Heidelberg University, Mannheim, Germany.
83 Beijing Institute of Ophthalmology, Beijing Tongren Hospital, Beijing, China.
84 Department of Nutrition and Dietetics, Mekelle University, Mekelle, Ethiopia.
85 Hematology-Oncology and Stem Cell Transplantation Research Center, Tehran University of Medical Sciences, Tehran, Iran.
86 Hematologic Malignancies Research Center, Tehran University of Medical Sciences, Tehran, Iran.
87 Department of Mental Health, University of Tokyo, Tokyo, Japan.
88 Department of Public Health and Community Medicine, Jordan University of Science and Technology, Ramtha, Jordan.
89 Social Determinants of Health Research Center, Ahvaz Jundishapur University of Medical Sciences, Ahvaz, Iran.
90 Epidemiology and Biostatistics Department, Health Services Academy, Islamabad, Pakistan.
91 International Otorhinolaryngology Research Association (IORA), Universal Scientific Education and Research Network (USERN), Tehran, Iran.
92 Department of Nutrition and Health Science, Ball State University, Muncie, IN, USA.
93 School of Medicine, Xiamen University Malaysia, Sepang, Malaysia.
94 Department of Nutrition, Simmons College, Boston, MA, USA.
95 Department of Health Management and Health Economics, Kristiania University College, Oslo, Norway.
96 Department of Health Services Policy and Management, University of South Carolina, Columbia, SC, USA.
97 Independent Consultant, Jakarta, Indonesia.
98 Department of Internal and Pulmonary Medicine, Sheri Kashmir Institute of Medical Sciences, Srinagar, India.
99 CIBERSAM, San Juan de Dios Sanitary Park, Sant Boi de Llobregat, Spain.
100 Catalan Institution for Research and Advanced Studies (ICREA), Barcelona, Spain.
101 Department of Demography, University of Montreal, Montreal, QC, Canada.
102 Department of Social and Preventive Medicine, University of Montreal, Montreal, QC, Canada.
103 Department of Public Health, Maragheh University of Medical Sciences, Maragheh, Iran.
104 Asbestos Diseases Research Institute, University of Sydney, Sydney, NSW, Australia.

105 Regional Centre for the Analysis of Data on Occupational and Work-related Injuries and Diseases, Local Health Unit Tuscany Centre, Florence, Italy.
106 Department of Health Sciences, University of Florence, Florence, Italy.
107 School of Public Health, University of Haifa, Haifa, Israel.
108 Institute of Medicine, Tribhuvan University, Kathmandu, Nepal.
109 Department of Public Health, Trnava University, Trnava, Slovakia.
110 Digestive Diseases Research Institute, Tehran University of Medical Sciences, Tehran, Iran.
111 Non-Communicable Diseases Research Center, Shiraz University of Medical Sciences, Shiraz, Iran.
112 Department of Epidemiology and Biostatistics, Tehran University of Medical Sciences, Tehran, Iran.
113 Campus Caucaia, Federal Institute of Education, Science and Technology of Ceará, Caucaia, Brazil.
114 Division of Plastic Surgery, University of Washington, Seattle, WA, USA.
115 Department of Internal Medicine, SevenHills Hospital, Mumbai, India.
116 Department of Medical Laboratory Science, Bahir Dar University, Bahir Dar, Ethiopia.
117 Research Department Prince Mohammed Bin Abdulaziz Hospital, Ministry of Health, Riyadh, Saudi Arabia.
118 College of Medicine, Alfaisal University, Riyadh, Saudi Arabia.
119 Peru Country Office, United Nations Population Fund (UNFPA), Lima, Peru.
120 Breast Surgery Unit, Helsinki University Hospital, Helsinki, Finland.
121 Clinical Microbiology and Parasitology Unit, Dr. Zora Profozic Polyclinic, Zagreb, Croatia.
122 University Centre Varazdin, University North, Varazdin, Croatia.
123 Global Institute of Public Health, Ananthapuri Hospitals and Research Institute, Trivandrum, India, Trivandrum, India.
124 Achutha Menon Centre for Health Science Studies, Sree Chitra Tirunal Institute for Medical Sciences and Technology, Trivandrum, India.
125 Faculty of General Medicine, Kyrgyz State Medical Academy, Bishkek, Kyrgyzstan.
126 Department of Atherosclerosis and Coronary Heart Disease, National Center of Cardiology and Internal Disease, Bishkek, Kyrgyzstan.
127 Heidelberg Institute of Global Health (HIGH), Faculty of Medicine and University Hospital, Heidelberg University, Heidelberg, Germany.
128 Institute of Addiction Research (ISFF), Frankfurt University of Applied Sciences, Frankfurt, Germany.
129 Department of Biostatistics, Hamadan University of Medical Sciences, Hamadan, Iran.
130 Health Systems and Policy Research Unit, Ahmadu Bello University, Zaria, Nigeria.
131 Clinical Epidemiology and Public Health Research Unit, Burlo Garofolo Institute for Maternal and Child Health, Trieste, Italy.
132 Department of Public Health Medicine, University of KwaZulu-Natal, Durban, South Africa.
133 Health Sciences Research Center, Mazandaran University of Medical Sciences, Sari, Iran.
134 Social Determinants of Health Research Center, Kurdistan University of Medical Sciences, Sanandaj, Iran.
135 Department of Epidemiology and Biostatistics, Kurdistan University of Medical Sciences, Sanandaj, Iran.
136 International Laboratory for Air Quality and Health, Queensland University of Technology, Brisbane, QLD, Australia.
137 Department of Surgery, University of Washington, Seattle, WA, USA.
138 Department of Health Management and Economics, Tehran University of Medical Sciences, Tehran, Iran.

139 Department of Pediatric Medicine, Nishtar Medical University, Multan, Pakistan.
140 Department of Pediatrics, Institute of Mother & Child Care, Multan, Pakistan.
141 Suraj Eye Institute, Nagpur, India.
142 Anatomy and Embryology Department, Carol Davila University of Medicine and Pharmacy, Bucharest, Romania.
143 Department of Cardiology, Cardio-Aid, Bucharest, Romania.
144 Centre of Cardiovascular Research and Education in Therapeutics, Monash University, Melbourne, VIC, Australia.
145 Translational Health Research Institute, Western Sydney University, Penrith, NSW, Australia.
146 Department of Psychiatry and Behavioural Neurosciences, McMaster University, Hamilton, ON, Canada.
147 Department of Psychiatry, University of Lagos, Lagos, Nigeria.
148 Centre for Healthy Start Initiative, Lagos, Nigeria.
149 Department of TB & Respiratory Medicine, Jagadguru Sri Shivarathreeswara University, Mysore, India.
150 Augenpraxis Jonas, Heidelberg University, Heidelberg, Germany.
151 Department of Medical Humanities and Social Medicine, Kosin University, Busan, South Korea.
152 Regional Medical Research Centre, Indian Council of Medical Research, Bhubaneswar, India.
153 Non-communicable Diseases Research Center, Alborz University of Medical Sciences, Karaj, Iran.
154 Department of Epidemiology & Biostatistics, Contech School of Public Health, Lahore, Pakistan.
155 Contech International Health Consultants, Lahore, Pakistan.
156 Department of Immunology, Mazandaran University of Medical Sciences, Sari, Iran.
157 Molecular and Cell Biology Research Center, Mazandaran University of Medical Sciences, Sari, Iran.
158 Thalassemia and Hemoglobinopathy Research Center, Ahvaz Jundishapur University of Medical Sciences, Ahvaz, Iran.
159 Endocrinology and Metabolism Molecular-Cellular Sciences Institute, Tehran University of Medical Sciences, Tehran, Iran.
160 Sina Trauma and Surgery Research Center, Tehran University of Medical Sciences, Tehran, Iran.
161 Department of Health Education & Promotion, Kermanshah University of Medical Sciences, Kermanshah, Iran.
162 Environmental Determinants of Health Research Center, Kermanshah University of Medical Sciences, Kermanshah, Iran.
163 Department of Clinical Research, Universidade Federal de Uberlândia, Uberlândia, Brazil.
164 Golestan Research Center of Gastroenterology and Hepatology, Golestan University of Medical Sciences, Gorgan, Iran.
165 Medical Department, University of Sharjah, Sharjah, United Arab Emirates.
166 Department of Epidemiology and Biostatistics, School of Public Health, Tabriz University of Medical Sciences, Tabriz, Iran.
167 Multiple Sclerosis Research Center, Tehran University of Medical Sciences, Tehran, Iran.
168 Department of Entomology, Ain Shams University, Cairo, Egypt.
169 Department of Psychology, University of Alabama at Birmingham, Birmingham, AL, USA.
170 Environmental Health Associates LLC, Englewood, CO, USA.

171 School of Public Health, University of Colorado Denver, Denver, CO, USA.
172 Independent Consultant, Karachi, Pakistan.
173 Usher Institute of Population Health Sciences and Informatics, University of Edinburgh, Edinburgh, UK.
174 Division of General Internal Medicine and Primary Care, Harvard University, Boston, MA, USA.
175 National Institute of Infectious Diseases, Tokyo, Japan.
176 Finnish Institute of Occupational Health, Helsinki, Finland.
177 Cancer Research Institute, Tehran University of Medical Sciences, Tehran, Iran.
178 Cancer Biology Research Center, Tehran University of Medical Sciences, Tehran, Iran.
179 UCIBIO, University of Porto, Porto, Portugal.
180 Department of Epidemiology, School of Preventive Oncology, Patna, India.
181 Social Development and Health Promotion Research Center, Kermanshah University of Medical Sciences, Kermanshah, Iran.
182 Hospital Universitario de la Princesa, Universidad Autónoma de Madrid, Madrid, Spain.
183 Centro de Investigación en Red de Enfermedades Respiratorias (CIBERES), Instituto de Salud Carlos III (ISCIII), Madrid, Spain.
184 Division of Community Medicine, International Medical University, Kuala Lumpur, Malaysia.
185 Department of Psychology, Deakin University, Burwood, VIC, Australia.
186 Department of Community Medicine, Ahmadu Bello University, Zaria, Nigeria.
187 Department of Medicine, University of Valencia, Valencia, Spain.
188 Carlos III Health Institute, Biomedical Research Networking Center for Mental Health Network (CiberSAM), MADRID, Spain.
189 School of Public Health, University of Adelaide, Adelaide, SA, Australia.
190 Department of Environmental Health, Wollo University, Dessie, Ethiopia.
191 Department of Pediatrics, King Saud University, Riyadh, Saudi Arabia.
192 Department of Pathology and Legal Medicine, University of São Paulo, Ribeirão Preto, Brazil.
193 Department of Health Economics, Hanoi Medical University, Hanoi, Vietnam.
194 Department of Molecular Medicine and Pathology & Auckland Cancer Society Research Centre, University of Auckland, Auckland, New Zealand.
195 Maurice Wilkins Centre for Biodiscovery, Auckland, New Zealand.
196 Lee Kong Chian School of Medicine, Nanyang Technological University, Singapore, Singapore.
197 Gomal Center of Biochemistry and Biotechnology, Gomal University, Dera Ismail Khan, Pakistan.
198 TB Culture Laboratory, Mufti Mehmood Memorial Teaching Hospital, Dera Ismail Khan, Pakistan.
199 Argentine Society of Medicine, Buenos Aires, Argentina.
200 Hospital Velez Sarsfield, Buenos Aires, Argentina.
201 University Medical Center Groningen, University of Groningen, Groningen, Netherlands.
202 Department of General Practice, University Medical Center Groningen, Groningen, Netherlands.
203 UKK Institute, Tampere, Finland.
204 Department of Medical and Surgical Sciences, University of Bologna, Bologna, Italy.
205 Occupational Health Unit, Sant’Orsola Malpighi Hospital, Bologna, Italy.
206 Department of Environmental Health, Harvard University, Boston, MA, USA.
207 Foundation University Medical College, Foundation University Islamabad, Rawalpindi, Pakistan.

208 Department of Psychiatry, University of São Paulo, São Paulo, Brazil.
209 University of South Australia, Adelaide, NSW, Australia.
210 Department of Psychopharmacology, National Center of Neurology and Psychiatry, Tokyo, Japan.
211 Department of Epidemiology and Biostatistics, Wuhan University, Wuhan, China.
212 Global Health Institute, Wuhan University, Wuhan, China.
213 Student Research Committee, Babol University of Medical Sciences, Babol, Iran.
214 Department of Global Health, Emory University, Atlanta, Georgia, USA
215 ISGlobal, Barcelona, Spain
